# Examining the interconnections among income, food prices, food insecurity, and health expenditure: a multicausality approach

**DOI:** 10.1186/s12889-025-24153-6

**Published:** 2025-08-14

**Authors:** Ahmet Murat Günal, Sevde Cantürk, Salim Yılmaz, Canser Boz, Derya Karabay

**Affiliations:** 1 Department of Nutrition and Dietetics, Faculty of Health Sciences, Halic University, Istanbul, 34060 Türkiye; 2 Department of Nutrition and Dietetics, Faculty of Health Sciences, Acibadem Mehmet Ali Aydinlar University, Istanbul, 34752 Türkiye; 3 Department of Health Management, Faculty of Health Sciences, Acibadem Mehmet Ali Aydinlar University, Istanbul, 34752 Türkiye; 4 Department of Health Management, Faculty of Health Sciences, Istanbul University-Cerrahpaşa, Istanbul, 34555 Türkiye; 5 Department of Health Management, Faculty of Health Sciences, Istanbul Arel University, Istanbul, 54187 Türkiye

**Keywords:** Food insecurity, Food prices, Income inequality, Health expenditure, Causal relationships

## Abstract

**Background:**

Food insecurity is a growing global issue driven by income inequality, food price fluctuations, and unequal access to essential resources. However, the interrelations among income distribution, real income, food prices, food insecurity, and health expenditure are not well understood, especially in terms of indirect and mediated effects.

**Materials and methods:**

We used a longitudinal dataset to build vector autoregression models and applied the Toda–Yamamoto causality approach to examine direct and mediated pathways. The augmented Dickey–Fuller test assessed stationarity, and optimal lag lengths were selected using the Akaike information criterion. We used the K-means algorithm for income group classification and the Wald test for comparing findings across groups, based on data from 99 countries. Structural stability was tested using CUSUM test for parameter stability and CUSUMSQ test for variance stability of recursive residuals and Bai–Perron for multiple breakpoints.

**Results:**

Income distribution and real income directly influenced food prices. In turn, food prices significantly impacted both food insecurity and health expenditure. In high-income countries, food insecurity was found to play a partial mediating role in the relationship between food prices and health expenditure. Importantly, in the global sample, income inequality mediated the relationship between food prices and food insecurity. The joint analysis of all variables revealed causal pathways that were not evident in isolated models.

**Conclusion:**

These findings highlight the critical role of income inequality in worsening food insecurity and increasing health burdens. The observed mediation mechanisms also suggest that targeted, income group–specific interventions are needed to effectively mitigate the compounded impacts of food inflation on health systems. Addressing economic disparities, stabilizing food prices, and enhancing welfare systems could reduce both food-related and healthcare challenges. Future research should explore regional patterns and broader socioeconomic indicators to support sustainable policy design.

**Supplementary Information:**

The online version contains supplementary material available at 10.1186/s12889-025-24153-6.

## Introduction

Food insecurity is defined as “a household-level economic and social condition of limited or uncertain access to adequate food” by the U.S. Department of Agriculture [1, 2]. In broader global terms, it reflects a lack of physical, social, and economic access to sufficient, safe, and nutritious foods required to lead an active and healthy life. According to the State of Food Security and Nutrition in the World 2024 report, the number of people facing hunger increased by approximately 152 million from 2019 to 2023, reaching 733 million globally, while over 2.3 billion people experienced moderate to severe food insecurity [3]. One of the main drivers of this increase is a sharp increase in global food prices.

Recent economic crises, such as the COVID-19 pandemic and the Ukraine–Russia war, have aggravated the rise in food prices [4]. According to the Food Price Index report of the FAO, the price of grains and vegetable oils have increased by more than 40% since 2014–2016 [5]. These fluctuations have severely constrained access to essential food items, especially for low-income populations in low- and middle-income countries, leading to increased food insecurity and negative health consequences globally [3]. For instance, a study analyzing data from 31 Sub-Saharan African countries between 2001 and 2018 demonstrated that a 1% increase in undernutrition rates reduced life expectancy by 0.00348% points and increased infant mortality by 0.0119% points, whereas a 1% increase in average daily energy intake decreased infant mortality by 0.0139% points [6]. Similarly, evidence from the United States indicates that rising food prices significantly exacerbate food insecurity and related adverse health outcomes [7, 8]. Collectively, these findings underscore the interconnectedness of food price volatility, food insecurity, and public health challenges across diverse socioeconomic contexts.

Food insecurity is not driven by rising food prices alone but also by income inequality, economic growth rate, and the structural economic imbalances they collectively create. The unequal distribution of economic growth has widened income disparities and further exacerbated economic inequalities [9]. Economic growth without equitable wealth distribution makes low-income groups highly vulnerable to rising living costs, compromising access to quality food, which leads to malnutrition and widespread food insecurity [10].

Food insecurity has far-reaching, multidimensional impacts on physical and mental health, which makes it a key factor in rising healthcare expenditures. It is strongly associated with all forms of malnutrition, including stunted growth, wasting, hidden hunger, and obesity [11]. Limited access to healthy and nutritious foods substantially increases the risk of noncommunicable diseases (NCDs), particularly diabetes, hypertension, cardiovascular diseases, and metabolic syndrome, all of which lead to a high healthcare burden [12, 13].

Understanding the direct and indirect relationships between health expenditure and food insecurity is key to ensure the long-term sustainability of healthcare systems. Nutritional deficiencies and diet-related diseases considerably increase individual healthcare costs and public health budgets. According to Tarasuk et al., the healthcare expenditure of households experiencing severe food insecurity is 75% higher than those of food-secure households [14].

While individual effects of food insecurity and economic disparities on health outcomes are known, the complex interplay among food prices, food insecurity, per capita gross domestic product at purchasing power parity (GDP (PPP)), the Gini coefficient, and health expenditure remains underexplored. By using a multicausality modeling approach, this study determines whether food price fluctuations drive food insecurity and increased health expenditures and whether their combined effects, mediated by economic inequality and income level, increase healthcare costs far beyond their individual influences.

## Materials and methods

This study examined the direct and potentially indirect causal relationships among key global economic and social variables, namely, the FAO’s global Food Price Index (*FPI*; as a proxy for food prices) the World Bank’s Prevalence of Severe Food Insecurity Index (*FI*; as a proxy for food insecurity), per capita gross domestic product in purchasing power parity terms (*GDP_PPP;* as a proxy for real income level), per capita health expenditure (*HE*), and the Gini coefficient (*GINI*; as a proxy for income inequality). Both developed and developing countries exhibit considerable fluctuations in food prices and differences in household income level, which has important implications for food insecurity, health outcomes, and broader economic inequality. Therefore, this study utilized global data and did not limit itself to merely identifying the univariate effects. It constructed multiple causality models to determine whether some variables exhibit indirect effects when analyzed with additional explanatory factors. Doing so reveals the intermediary or joint effects that may be overlooked by a classical single-equation vector autoregression (VAR) model. This study did not focus solely on a linear model involving one causal variable and one outcome variable. It also investigated whether multiple independent variables collectively influence a dependent variable, which is crucial to determine whether causality is stronger when the variables act together rather than separately. Through this multifaceted approach, the study provides a more comprehensive understanding of whether a variable indirectly affects another—for instance, whether a variable exerts influence through an intermediary. Considering the potential bidirectional and time-lagged relationships among the selected variables—such as the reciprocal nature of food insecurity and health expenditure—the use of simple unidirectional models was deemed insufficient. Instead, the Toda–Yamamoto causality framework was adopted as it can accommodate lagged and possibly endogenous dynamics while focusing on the directionality of causality. This choice aligns with the study’s primary aim, which is not to estimate the magnitude of effects per unit but to determine whether a variable exerts a statistically meaningful direct or indirect causal influence through mediation or joint pathways.

The theoretical framework of the baseline model was designed to examine the potential causal effects of each variable on another, directly and indirectly (through mediation). First, we investigated the causal effects of two critical economic indicators—*GINI* and *GDP_PPP*—on *FPI* (Models 1 and 2). The literature has shown that income inequality and food prices can influence each other and that income level—particularly when adjusted for real value and purchasing power parity—plays an important role in price fluctuations [15–17].

Next, we tested the direct effects of *GINI* and *GDP_PPP* on *FI* (Models 3 and 4) to examine how inequality and real income growth influence societal access to food. Additionally, Models 5 and 6 were constructed to determine whether both *GINI* and *GDP_PPP* have an indirect effect on *FI* through *FPI*.

**Fig. 1:**
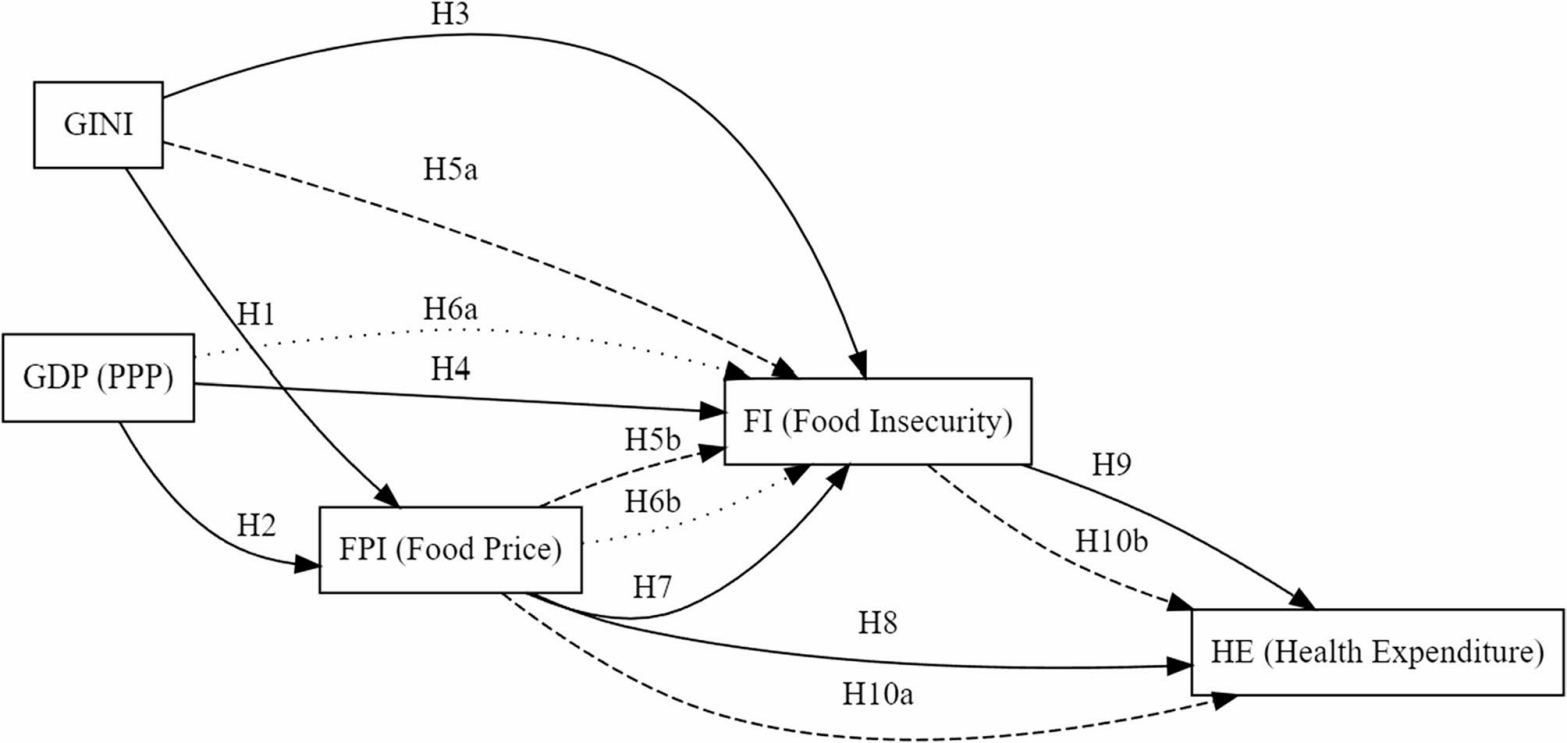
Theoretical model

In this setup, where *GINI* and *FPI* jointly influence *FI* or *GDP_PPP* and *FPI* jointly influence *FI*, we investigated whether multiple explanatory variables exert combined and/or indirect effects on one dependent variable (*FI*). Similarly, the independent effect of *FPI* on *FI* was tested separately (Model 7). The literature has provided robust evidence that food prices can directly trigger food insecurity [6, 18–20]. Next, we examined the effects of *FPI* on *HE* (Model 8) and *FI* on *HE* (Model 9) to include the relationship with health expenditure. Additionally, we assessed the joint effect of *FPI* and *FI* on *HE* (Model 10) to determine whether *FPI* influences *HE* through *FI*. As illustrated in Fig. 1, these relationships are captured in the theoretical baseline model.

In cross-sectional studies, the Baron and Kenny [8] approach is predominantly used to evaluate mediating effects [21, 22]. We used the causal adaptation of the same approach. In a multiple causal approach, the singular effect of one variable that may be a mediator is examined. However, for a variable to be considered a mediator in a cross-sectional study, the independent variable must significantly affect both the mediating variable and the dependent variable [21, 23]. Although the VAR model reveals mutual dynamic interactions, the Toda–Yamamoto approach, which is an extension of the conventional Granger causality framework, allows the examination of unidirectional effects. If it is confirmed that the causal variable has a significant effect on the mediator variable, that the causal variable directly affects the outcome variable, and that the mediator variable affects the outcome variable in a multiple model, then we consider an indirect causal effect to be present. This approach can be considered a longitudinal causal adaptation of the Baron and Kenny approach [24, 25]. Furthermore, the multiple causal approach reveals how the interaction between *FPI* and *FI* reflects on *HE*, specifically, whether both variables work together or if one acts as an indirect mediator. Therefore, this design constitutes a comprehensive and systematic causality analysis framework that uncovers univariate direct effects and whether one or more variables together have an indirect causal effect.

Data for the variables used in the study, shown in Table 1, were drawn from the FAO, World Bank, World Inequality Database (WID), and Economist Impact, covering different periods between 1980 and 2024. The food-insecurity variable (FI) was assembled at the global level by harmonizing FAO’s Prevalence of Undernourishment series from the State of Food Insecurity in the World reports (SOFI) (2000–2014), with FAO’s FIES-based Prevalence of Severe Food Insecurity data disseminated via Our World in Data (2015–2022), and the FI time series is openly archived on Zenodo with methodological notes [26]. Because FI data could not be disaggregated by income group, we included the Global Food Security Index (FS), obtained directly from Economist Impact’s annual datasets (2012–2022), as a complementary, index-based, country-level measure to enable income-stratified comparisons. Furthermore, because the FAO global Food Price Index (FPI)—which is indexed to the 2014–2016 average (= 100)—does not provide country-level data, we additionally incorporated Consumer Price Index– Food (CPI) data (2012–2022), sourced from the FAO Consumer Prices, Food Indices dataset (base year 2015 = 100), to enable more granular analyses of food price dynamics across income groups. Both indices (FPI and CPI) are already published in real terms by FAO and were used directly without transformation; GDP per capita at purchasing power parity (GDP_PPP) was employed to control for cross-country differences in price levels. Initially, as the 2022 health expenditure data were not yet released by the World Bank, we provisionally imputed this observation using Holt’s linear trend, evaluating model fit with mean absolute percentage error (MAPE) and root mean square error (RMSE); once the official 2022 figure became available, it was incorporated into the final dataset, and all models were re-estimated accordingly, with final analyses based on the officially published values.

**Table 1:** Raw variables used

Code	Variable name	Source	Years	Access
*FPI*	Food Price Index (annual)	FAO	1990–2024	Food and Agriculture Organization of the United Nations (2023). Availability (based on supply utilization accounts) https://www.fao.org/faostat/en/#data/SUA, accessed on January 9, 2025.
*CPI*	Consumer Price Index– Food (annual)	FAO	2012–2022	Food and Agriculture Organization of the United Nations (2025). Consumer Price Indices, Food (2015 = 100), https://www.fao.org/faostat/en/#data/CP, accessed on June 21, 2025.
*FI*	Prevalence of Severe Food Insecurity (%)	FAO	2000–2022	World Food Insecurity Dataset (2000–2022), Harmonized from FAO SOFI and FIES-based Indicators, https://zenodo.org/records/15712022, accessed on 21 June 2025.
*FS*	Global Food Security Index	Economist Impact	2012–2022	Economist Impact (2023). Global Food Security Index, 2022. https://impact.economist.com/sustainability/project/food-security-index/download-the-index, accessed on June 21, 2025.
*GDP_PPP*	GDP (per capita, PPP)	World Bank	1990–2023	World Bank (2023). GDP at PPP (constant 2021 international USD), (2024). https://data.worldbank.org/indicator/NY.GDP.MKTP.CD, accessed on January 29, 2025.
*HE*	Health expenditure ($, PPP)	World Bank	2000–2022	World Bank (2024). Current health expenditure (USD, PPP). https://data.worldbank.org/indicator/SH.XPD.CHEX.PC.CD?locations=1 W, accessed on January 9, 2025.
*GINI*	The GINI coefficient (per capita)	The World Inequality Database	1980–2022	World Inequality Database (2024). Income inequality, World 1980–2022. World Inequality Database (WID), https://wid.world, accessed on January 29, 2025.

Stationarity analyses were first conducted for each series using the augmented Dickey–Fuller (ADF) test to determine whether the variables are stationary at their levels or in differenced forms. Next, the optimal lag lengths for different VAR models were identified using the Akaike information criterion (AIC). Variance inflation factor (VIF) values were calculated to address potential multicollinearity among the independent variables in multiple models. Finally, the models were constructed using the Toda–Yamamoto causality approach.

Toda and Yamamoto’s (1995) method more flexibly addresses the stationarity and cointegration requirements of the traditional Granger causality analysis [27] in which variables must be integrated at the same order (e.g., all I(1)) or cointegration relationships must be identified, otherwise model misspecification or spurious regression may occur. However, in the Toda–Yamamoto approach, the model is constructed as VAR(*p* + *dmax*), which allows the use of variables at their levels. Here, *p* represents the optimal lag length selected based on the information criteria, while *dmax* denotes the highest order of integration among the series. This approach ensures that the test results remain valid even if the series are not entirely stationary in their levels, unlike in the traditional Granger procedure. By adding an extra lag to the VAR model, the approach effectively mitigates problems arising from the integration order of the series [28].

When using the Toda–Yamamoto approach, after estimating the coefficients of the constructed VAR(*p* + *d_max*) model, the Wald test is conducted only for *p* lags (i.e., the initially selected optimal lag length) to determine the direction of causality. The hypothesis tested here is whether the selected variables jointly have zero effect on the future values of the dependent variable. If the null hypothesis that the estimated coefficients are jointly equal to zero is rejected through the Wald test, it can be concluded that the specified direction of causality exists. The advantage of this method is that it allows for causality analysis to be conducted directly on the level values of the series, even if they have different integration orders (I(0), I(1),…, etc.) or if the cointegration relationships are not explicitly identified. In this respect, the Toda–Yamamoto approach is appropriate because it makes the assumptions relatively easier to satisfy and helps prevent information loss because of differencing, particularly for long-term relationships [27, 28].

Rather than examining the effect of one causal variable on one outcome variable, multiple explanatory (causal) variables can be included in the same equation to explore their joint and individual contributions to a dependent variable. For this purpose, a VAR(*p* + *d_max*) model is constructed again. However, when testing causality, the main focus is whether the lagged coefficients of the multiple variables in the equation are jointly equal to zero.

This approach can be used to examine the combined (total) effect of *GINI* and *FPI* on *FI*, followed by their individual contributions using a submodel (e.g., examining only *GINI* or *FPI*). If a variable that is nonsignificant on its own becomes significant when analyzed with another variable, this may indicate an indirect or a joint effect. This, in turn, allows the exploration of mediation or interaction possibilities. Using the Toda–Yamamoto method in a multiple-model framework is more appropriate for detecting complex causal patterns—particularly indirect (mediated) effects and joint (combined) effects—than traditional single-variable causality analyses [24, 25, 27]. Therefore, we propose the following alternative hypotheses:

- H_1_: Income distribution has a significant causal effect on food prices.
- H_2_: Real income has a significant causal effect on food prices.
- H_3_: Income distribution has a significant causal effect on food insecurity.
- H_4_: Real income has a significant causal effect on food insecurity.
- H_5_: Income distribution and food prices together have a significant causal effect on food insecurity.
  - H_5a_: When considered together, income distribution has a significant individual causal effect on food insecurity.
  - H_5b_: When considered together, food prices have a significant individual causal effect on food insecurity.
- H_6_: Real income and food prices together have a significant causal effect on food insecurity.
  - H_6a_: When considered together, real income has a significant individual causal effect on food insecurity.
  - H_6b_: When considered together, food prices have a significant individual causal effect on food insecurity.
- H_7_: Food prices have a significant causal effect on food insecurity.
- H_8_: Food prices have a significant causal effect on health expenditure.
- H_9_: Food insecurity has a significant causal effect on health expenditure.
- H_10_: Food prices and food insecurity together have a significant causal effect on health expenditure.
  - H_10a_: When considered together, food prices have a significant individual causal effect on health expenditure
  - H_10b_: When considered together, food insecurity has a significant individual causal effect on health expenditure.

Based on these hypotheses, the baseline model equations are formulated as follows:

$$\begin{aligned} \:{FPI}_{t}=&{\beta\:}_{0}+\sum\:_{i=1}^{k}{\alpha\:}_{1i}*{FPI}_{t-i}\\&+\sum\:_{j=k+1}^{k+dmax}{\beta\:}_{1j}*{FPI}_{t-j}\\&+\sum\:_{j=1}^{k}{\alpha\:}_{2i}*{GINI}_{t-i}\\&+\sum\:_{j=k+1}^{k+dmax}{\theta\:}_{2j}*{GINI}_{t-j}\\&+{\epsilon\:}_{1t}\:for\:[H1-Hypotheses\:\alpha\:] \end{aligned}$$

$$\begin{aligned} \:{FPI}_{t}=&{\beta\:}_{0}+\sum\:_{i=1}^{k}{\alpha\:}_{1i}*{FPI}_{t-i}\\&+\sum\:_{j=k+1}^{k+dmax}{\beta\:}_{1j}*{FPI}_{t-j}\\&+\sum\:_{i=1}^{k}{\alpha\:}_{2i}*{GDP}_{t-i}\\&+\sum\:_{j=k+1}^{k+dmax}{\theta\:}_{2j}*{GDP}_{t-j}\\&+{\epsilon\:}_{1t}\:for\:[H2-Hypotheses\:\alpha\:] \end{aligned}$$

$$\begin{aligned} \:{FI}_{t}=&{\beta\:}_{0}+\sum\:_{i=1}^{k}{\alpha\:}_{1i}*{FI}_{t-i}\\&+\sum\:_{j=k+1}^{k+dmax}{\beta\:}_{1j}*{FI}_{t-j}\\&+\sum\:_{i=1}^{k}{\alpha\:}_{2i}*{GINI}_{t-i}\\&+\sum\:_{j=k+1}^{k+dmax}{\theta\:}_{2j}*{GINI}_{t-j}\\&+{\epsilon\:}_{1t}\:for\:[H3-Hypotheses\:\alpha\:] \end{aligned}$$

$$\begin{aligned} \:{FI}_{t}=&{\beta\:}_{0}+\sum\:_{i=1}^{k}{\alpha\:}_{1i}*{FI}_{t-i}\\&+\sum\:_{j=k+1}^{k+dmax}{\beta\:}_{1j}*{FI}_{t-j}\\&+\sum\:_{i=1}^{k}{\alpha\:}_{2i}*{GDP}_{t-i}\\&+\sum\:_{j=k+1}^{k+dmax}{\theta\:}_{2j}*{GDP}_{t-j}\\&+{\epsilon\:}_{1t}\:for\:[H4-Hypotheses\:\alpha\:] \end{aligned}$$

$$\begin{aligned} \:{FI}_{t}=&{\beta\:}_{0}+\sum\:_{i=1}^{k}{\eta\:}_{1i}*{FI}_{t-i}\\&+\sum\:_{j=k+1}^{k+dmax}{\vartheta\:}_{1j}*{FI}_{t-j}\\&+\sum\:_{i=1}^{k}{\alpha\:}_{2i}*{GINI}_{t-i}\\&+\sum\:_{j=k+1}^{k+dmax}{\theta\:}_{2j}*{GINI}_{t-j}\\&+\sum\:_{i=1}^{k}{\alpha\:}_{2i}*{FPI}_{t-i}\\&+\sum\:_{j=k+1}^{k+dmax}{\theta\:}_{2j}*{FPI}_{t-j}\\&+{\epsilon\:}_{1t}\:for\:[H5-Hypotheses\:\alpha\:] \end{aligned}$$

$$\begin{aligned} \:{FI}_{t}=&{\beta\:}_{0}+\sum\:_{i=1}^{k}{\psi\:}_{1i}*{FI}_{t-i}\\&+\sum\:_{j=k+1}^{k+dmax}{\xi\:}_{1j}*{FI}_{t-j}\\&+\sum\:_{i=1}^{k}{\rho\:}_{2i}*{FPI}_{t-i}\\&+\sum\:_{j=k+1}^{k+dmax}{\lambda\:}_{2j}*{FPI}_{t-j}\\&+\sum\:_{i=1}^{k}{\varphi\:}_{2i}*{GINI}_{t-i}\\&+\sum\:_{j=k+1}^{k+dmax}{\varsigma\:}_{2j}*{GINI}_{t-j}+\\&+{\epsilon\:}_{1t}\:for\:[H5a-Hypotheses\:\alpha\:] \end{aligned}$$

$$\begin{aligned} \:{FI}_{t}=&{\beta\:}_{0}+\sum\:_{i=1}^{k}{\psi\:}_{1i}*{FI}_{t-i}\\&+\sum\:_{j=k+1}^{k+dmax}{\xi\:}_{1j}*{FI}_{t-j}\\&+\sum\:_{i=1}^{k}{\varphi\:}_{2i}*{GINI}_{t-i}\\&+\sum\:_{j=k+1}^{k+dmax}{\varsigma\:}_{2j}*{GINI}_{t-j}\\&+\sum\:_{i=1}^{k}{\rho\:}_{2i}*{FPI}_{t-i}\\&+\sum\:_{j=k+1}^{k+dmax}{\lambda\:}_{2j}*{FPI}_{t-j}\\&+{\epsilon\:}_{1t}\:for\:[H5b-Hypotheses\:\alpha\:] \end{aligned}$$

$$\begin{aligned} \:{FI}_{t}=&{\beta\:}_{0}+\sum\:_{i=1}^{k}{\eta\:}_{1i}*{FI}_{t-i}\\&+\sum\:_{j=k+1}^{k+dmax}{\vartheta\:}_{1j}*{FI}_{t-j}\\&+\sum\:_{i=1}^{k}{\alpha\:}_{2i}*{GDP}_{t-i}\\&+\sum\:_{j=k+1}^{k+dmax}{\theta\:}_{2j}*{GDP}_{t-j}\\&+\sum\:_{i=1}^{k}{\alpha\:}_{2i}*{FPI}_{t-i}\\&+\sum\:_{j=k+1}^{k+dmax}{\theta\:}_{2j}*{FPI}_{t-j}\\&+{\epsilon\:}_{1t\:}for\:[H6-Hypotheses\:\alpha\:]\end{aligned}$$

$$\begin{aligned} \:{FI}_{t}=&{\beta\:}_{0}+\sum\:_{i=1}^{k}{\psi\:}_{1i}*{FI}_{t-i}\\&+\sum\:_{j=k+1}^{k+dmax}{\xi\:}_{1j}*{FI}_{t-j}\\&+\sum\:_{i=1}^{k}{\rho\:}_{2i}*{FPI}_{t-i}\\&+\sum\:_{j=k+1}^{k+dmax}{\lambda\:}_{2j}*{FPI}_{t-j}\\&+\sum\:_{i=1}^{k}{\varphi\:}_{2i}*{GDP}_{t-i}\\&+\sum\:_{j=k+1}^{k+dmax}{\varsigma\:}_{2j}*{GDP}_{t-j}+\\&+{\epsilon\:}_{1t}\:for\:[H6a-Hypotheses\:\alpha\:] \end{aligned}$$

$$\begin{aligned} \:{FI}_{t}=&{\beta\:}_{0}+\sum\:_{i=1}^{k}{\psi\:}_{1i}*{FI}_{t-i}\\&+\sum\:_{j=k+1}^{k+dmax}{\xi\:}_{1j}*{FI}_{t-j}\\&+\sum\:_{i=1}^{k}{\varphi\:}_{2i}*{GDP}_{t-i}\\&+\sum\:_{j=k+1}^{k+dmax}{\varsigma\:}_{2j}*{GDP}_{t-j}\\&+\sum\:_{i=1}^{k}{\rho\:}_{2i}*{FPI}_{t-i}\\&+\sum\:_{j=k+1}^{k+dmax}{\lambda\:}_{2j}*{FPI}_{t-j}\\&+{\epsilon\:}_{1t}\:for\:[H6b-Hypotheses\:\alpha\:] \end{aligned}$$

$$\begin{aligned} \:{FI}_{t}=&{\beta\:}_{0}+\sum\:_{i=1}^{k}{\alpha\:}_{1i}*{FI}_{t-i}\\&+\sum\:_{j=k+1}^{k+dmax}{\beta\:}_{1j}*{FI}_{t-j}\\&+\sum\:_{i=1}^{k}{\alpha\:}_{2i}*{FPI}_{t-i}\\&+\sum\:_{j=k+1}^{k+dmax}{\theta\:}_{2j}*{FPI}_{t-j}\\&+{\epsilon\:}_{1t}\:for\:[H7-Hypotheses\:\alpha\:] \end{aligned}$$

$$\begin{aligned} \:{HE}_{t}=&{\beta\:}_{0}+\sum\:_{i=1}^{k}{\alpha\:}_{1i}*{HE}_{t-i}\\&+\sum\:_{j=k+1}^{k+dmax}{\beta\:}_{1j}*{HE}_{t-j}\\&+\sum\:_{i=1}^{k}{\alpha\:}_{2i}*{FPI}_{t-i}\\&+\sum\:_{j=k+1}^{k+dmax}{\theta\:}_{2j}*{FPI}_{t-j}\\&+{\epsilon\:}_{1t}\:for\:[H8-Hypotheses\:\alpha\:] \end{aligned}$$

$$\begin{aligned} \:{HE}_{t}=&{\beta\:}_{0}+\sum\:_{i=1}^{k}{\alpha\:}_{1i}*{HE}_{t-i}\\&+\sum\:_{j=k+1}^{k+dmax}{\beta\:}_{1j}*{HE}_{t-j}\\&+\sum\:_{i=1}^{k}{\alpha\:}_{2i}*{FI}_{t-i}\\&+\sum\:_{j=k+1}^{k+dmax}{\theta\:}_{2j}*{FI}_{t-j}\\&+{\epsilon\:}_{1t}\:for\:[H9-Hypotheses\:\alpha\:] \end{aligned}$$

$$\begin{aligned}\:{HE}_{t}=&{\beta\:}_{0}+\sum\:_{i=1}^{k}{\eta\:}_{1i}*{HE}_{t-i}\\&+\sum\:_{j=k+1}^{k+dmax}{\vartheta\:}_{1j}*{HE}_{t-j}\\&+\sum\:_{i=1}^{k}{\alpha\:}_{2i}*{FPI}_{t-i}\\&+\sum\:_{j=k+1}^{k+dmax}{\theta\:}_{2j}*{FPI}_{t-j}\\&+\sum\:_{i=1}^{k}{\alpha\:}_{2i}*{FI}_{t-i}\\&+\sum\:_{j=k+1}^{k+dmax}{\theta\:}_{2j}*{FI}_{t-j}\\&+{\epsilon\:}_{1t}\:for\:[H10-Hypotheses\:\alpha\:] \end{aligned}$$

$$\begin{aligned} \:{HE}_{t}=&{\beta\:}_{0}+\sum\:_{i=1}^{k}{\psi\:}_{1i}*{HE}_{t-i}\\&+\sum\:_{j=k+1}^{k+dmax}{\xi\:}_{1j}*{HE}_{t-j}\\&+\sum\:_{i=1}^{k}{\rho\:}_{2i}*{FI}_{t-i}\\&+\sum\:_{j=k+1}^{k+dmax}{\lambda\:}_{2j}*{FI}_{t-j}\\&+\sum\:_{i=1}^{k}{\varphi\:}_{2i}*{FPI}_{t-i}\\&+\sum\:_{j=k+1}^{k+dmax}{\varsigma\:}_{2j}*{FPI}_{t-j}+\\&+{\epsilon\:}_{1t}\:for\:[H10a-Hypotheses\:\alpha\:] \end{aligned}$$

$$\begin{aligned} \:{HE}_{t}=&{\beta\:}_{0}+\sum\:_{i=1}^{k}{\psi\:}_{1i}*{HE}_{t-i}\\&+\sum\:_{j=k+1}^{k+dmax}{\xi\:}_{1j}*{HE}_{t-j}\\&+\sum\:_{i=1}^{k}{\varphi\:}_{2i}*{FPI}_{t-i}\\&+\sum\:_{j=k+1}^{k+dmax}{\varsigma\:}_{2j}*{FPI}_{t-j}\\&+\sum\:_{i=1}^{k}{\rho\:}_{2i}*{FI}_{t-i}\\&+\sum\:_{j=k+1}^{k+dmax}{\lambda\:}_{2j}*{FI}_{t-j}\\&+{\epsilon\:}_{1t}\:for\:[H10b-Hypotheses\:\alpha\:] \end{aligned}$$

To ensure the temporal robustness of the causality relationships explored in the study, structural stability tests were systematically applied to all estimated models. Establishing stability is essential because the presence of structural breaks may result in time-varying parameters, potentially leading to misleading or spurious causality inferences. For single-equation models, the Bai–Perron multiple breakpoint test was employed. This method enables the endogenous identification of multiple structural shifts in regression coefficients by iteratively testing for breakpoints using sequential F-statistics. However, as the Bai–Perron technique is not compatible with multivariate systems, it was not applicable to the VAR-based models with multiple endogenous predictors. Therefore, for Models 5, 6, and 9, which involve multiple explanatory variables, we employed recursive CUSUM and CUSUM of Squares (CUSUM-SQ) tests to assess parameter stability. These tests analyze whether the estimated parameters remain consistent over time by tracking cumulative changes in recursive residuals. Specifically, the CUSUM test is sensitive to shifts in the mean of the residuals, while the CUSUM-SQ test detects changes in variance, offering a comprehensive check on structural stability in multivariable contexts (the graphical outputs of all stability tests are provided in the Supplementary Appendix).

The country-level Food Price dataset published by the FAO from 2012 to 2022 was employed to facilitate income-based stratification of countries and corresponding model testing. Countries were matched across the relevant variables, resulting in a final sample of 99 countries with complete and overlapping data. Income-level analyses were subsequently performed separately for the subgroups derived from this sample.

Prior to model implementation, countries were classified into income groups using a K-means clustering algorithm, based on the most recent data available for 2022, specifically per capita GDP adjusted for purchasing power parity (GDP PPP) and health expenditure (HE). Before clustering, all variables were standardized, and the optimal number of clusters was determined using the within-cluster sum of squares (WSS) and Silhouette methods. For each value of k, 25 random initializations were performed, and Euclidean distance was used as the distance metric in the clustering process. As a result of the analysis, countries were divided into two groups: “low-income countries” and “higher-income countries.” Model estimations were conducted separately for each group, and differences between these groups were assessed using the Wald test.

Data processing, statistical analysis, and causality tests were conducted using R version 4.4.2. For the graphical visualizations, the Draw.io software and the DiagrammeR package were used. The vars package in R was used for constructing the VAR models and implementing the Toda–Yamamoto approach [27, 29, 30]. ADF and other unit root tests were performed using the urea package, which allows the examination of the stationarity levels of the variables [31]. The cluster package was used for k-means clustering analyses to classify countries according to income level [32]. All of the additional statistical analyses, such as diagnostic tests on VAR results and Wald tests, were conducted using the latest package [33]. All of the statistical evaluations were conducted at a 95% confidence level.

## Results

**Fig. 2:**
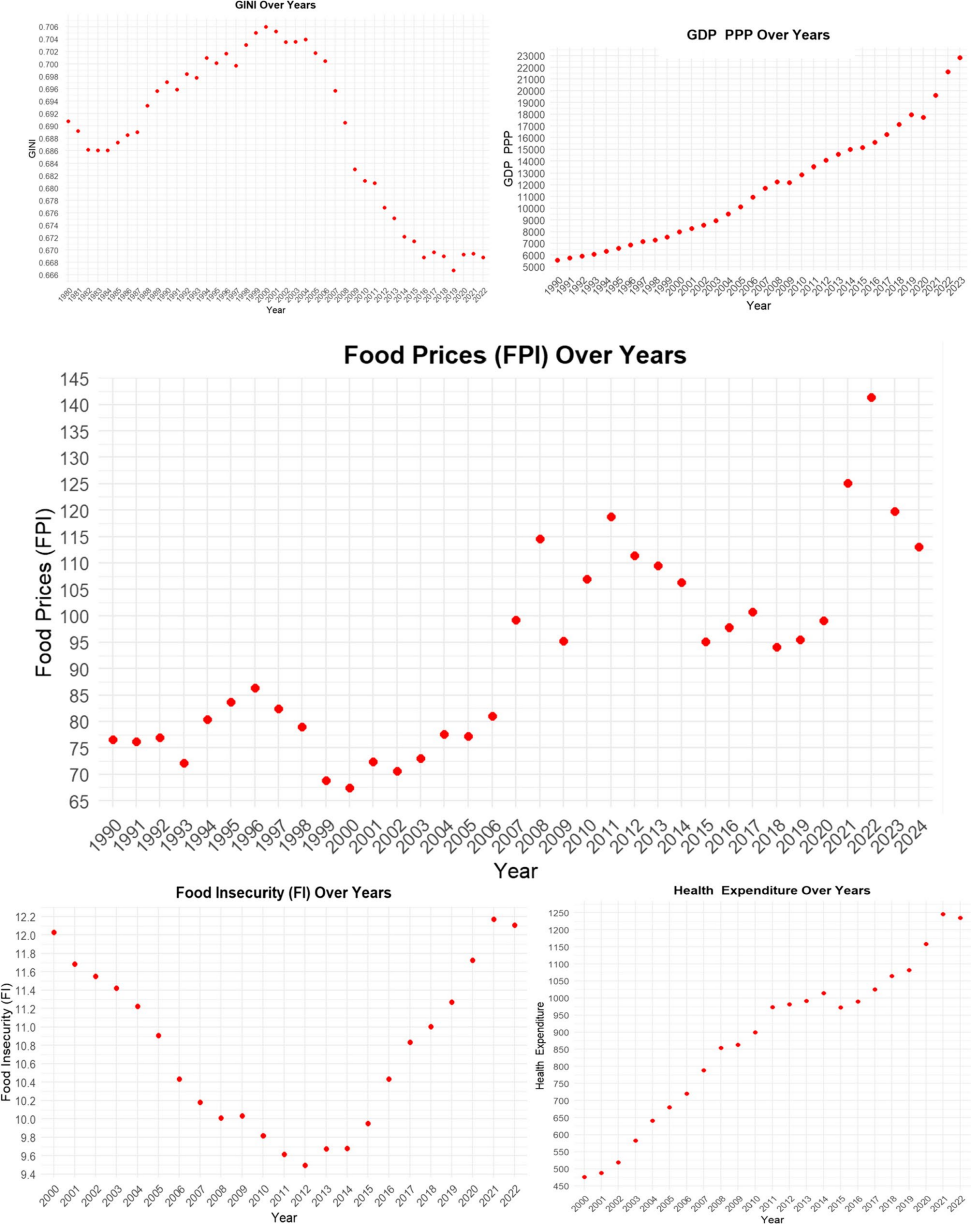
Food price index and other variables

Figure 2 presents the changes of the variables used in this study—*GINI*, *GDP_PPP*, *FPI*, *FI*, and *HE*—over the years.

Figure 2 shows that *GINI* values were moderate during the 1990 s, then started to increase during the early 2000 s and have been declining since the mid-2000s; this suggests that income inequality peaked during the early 2000 s and then gradually decreased. In contrast, the steady increase in *GDP_PPP* indicates that real income levels have significantly risen over time globally. Regarding *FPI*, there are periods in which nominal and real *FPI* diverge, with notable fluctuations, particularly during 2007–2008 and 2010–2011, which aligns with global economic crises in those years. The *FI* indicator was high in the early 2000 s, reached its lowest point around 2010, and has been increasing since then. Meanwhile, *HE* has risen almost consistently and rapidly since the 2000s.

Structural stability of the causality models was examined using the Bai–Perron multiple breakpoint test. Among the nine single-cause models tested, six exhibited statistically significant structural breaks (SupF test, *p* < 0.10). Specifically, FPI ← GINI (Model 1) showed one breakpoint in 2006 (*p* = 0.0888). FI ← GINI (Model 3) displayed multiple breakpoints in 2005, 2013, 2016, and 2019 (*p* = 0.0079). FI ← GDP_PPP (Model 4) had significant breakpoints in 2011, 2015, and 2019 (*p* = 0.0118). In the model HE ← FI (Model 9), breaks were detected in 2002, 2005, 2011, and 2016 (*p* = 0.0051). HE ← FPI (Model 8) exhibited breaks in 2004, 2008, 2011, 2016, and 2019 (*p* = 0.0099). Lastly, the model FPI ← FI (Model 7) showed structural changes in 2006 and 2019 (*p* = 0.0431).

CUSUM and CUSUM-SQ stability tests were applied to the multivariable VAR models to examine structural stability in the presence of multiple predictors. For the model FI ← GINI + FPI (Model 5), the CUSUM test yielded a statistically significant result (*p* = 0.0168), indicating instability in the regression structure over time. This finding was confirmed by the CUSUM-SQ test (*p* = 0.01), suggesting the presence of structural shifts. Similarly, for FI ← GDP per capita (PPP) + FPI (Model 6), both the CUSUM (*p* = 0.0088) and CUSUM-SQ (*p* = 0.01) tests indicated significant instability. In the final multivariable model, HE ← FI + FPI (Model 9), the CUSUM (*p* = 0.0357) and CUSUM-SQ (*p* = 0.0339) tests also revealed structural instability. These significant findings confirmed that the relationship structures were not stable over time in all tested multivariate VAR models.

**Table 2:** Determination of lag lengths and stationarity according to the models

VAR Model	AIC	Variable	t-value (%5)	*p*-value	Result
*GINI* → *FPI*	1	*GINI*	−2.283	0.0305*	S
		*FPI*	−2.109	0.0443*	S
*GDP_PPP* → *FPI*	4	*GDP_PPP*	0.147	0.884	NS
		*GDP_PPP* (Δ)	−4.028	0.0004***	S
		*FPI*	−2.909	0.00702**	S
*GINI* → *FI*	1	*GINI*	−0.86	0.402	NS
		*GINI* (Δ)	−2.194	0.0433*	S
		*FI*	−1.076	0.297	NS
		*FI* (Δ)	−2.785	0.0132*	S
*GDP_PPP* → *FI*	2	*GDP_PPP*	−0.925	0.368	NS
		*GDP_PPP* (Δ)	−2.755	0.0141*	S
		*FI*	−1.076	0.297	NS
		*FI* (Δ)	−2.785	0.0132*	S
*GINI* + *FPI* → *FI*	3_a_	*GINI*	−0.86	0.402	NS
		*GINI* (Δ)	−2.194	0.0433*	S
		*FPI*	−1.857	0.0808	NS
		*FPI* (Δ)	−3.037	0.00786**	S
		*FI*	−1.076	0.297	NS
		*FI* (Δ)	−2.785	0.0132**	S
*GDP_PPP* + *FPI* → *FI*	3	*GDP_PPP*	−0.925	0.368	NS
		*GDP_PPP* (Δ)	−2.755	0.0141**	S
		*FPI*	−1.856	0.0808	NS
		*FPI* (Δ)	−3.037	0.00786**	S
		*FI*	−1.076	0.297	NS
		*FI* (Δ)	−2.785	0.0132*	S
*FPI* → *FI*	3	*FPI*	−1.856	0.0808	NS
		*FPI* (Δ)	−3.037	0.00786**	S
		*FI*	−1.076	0.297	NS
		*FI* (Δ)	−2.785	0.0132*	S
*FPI* → *HE*	1	*FPI*	−1.856	0.0808	NS
		*FPI* (Δ)	−3.037	0.00786**	S
		*HE*	−2.083	0.0526	NS
		*HE* (Δ)	−3.541	0.0027**	S
*FI* → *HE*	5	*FI*	−1.076	0.297	NS
		*FI* (Δ)	−2.785	0.0132*	S
		*HE*	−2.083	0.0526	NS
		*HE* (Δ)	−3.541	0.0027**	S
*FI* + *FPI* → *HE*	1	*FI*	−1.076	0.297	NS
		*FI* (Δ)	−2.785	0.0132*	S
		*FPI*	−1.856	0.0808	NS
		*FPI* (Δ)	−3.037	0.00786**	S
		*HE*	−2.083	0.0526	NS
		*HE* (Δ)	−3.541	0.0027**	S

*NS* Nonstationary, *S* Stationary

*p < 0.05

**p < 0.01

***p < 0.001

a:maximum lag value is fixed at 3

Table 2 presents the ADF test results and the stationarity status of all the variables. The effect of *GINI* on *FPI* was stationary (S) at the level without first-order differencing, which indicates a statistically significant relationship between the two variables. However, the effect of *GDP_PPP* on *FPI* achieved stationarity only after first-order differencing. Similarly, the effect of *GINI* on *FI* and *GDP_PPP* on *FI* reached stationarity only after differencing. For the effect of *FI* on *HE*, both *FI* and *HE* became significant after differencing, and the effect of *FPI* on *HE* exhibited stationarity after first-order differencing. AIC values were used to determine the optimal lag length for the models. In the *GINI* → *FPI* and *GINI* → *FI* models, AIC = 1, which indicates that these variables have a short-term relationship. Similarly, in the *FPI* → *HE* and *FI* + *FPI* → *HE* models, AIC = 1. However, in the *GDP_PPP* → *FPI* model, AIC = 4, which indicates that the effect between the two variables emerges in the long term. For the *GDP_PPP* → *FI* model, AIC = 2, and for the *GDP_PPP* + *FPI* → *FI* model, AIC = 3, which indicates a medium-term effect. In contrast, in the *GINI* + *FPI* → *FI* and *FI* → *HE* models, AIC = 5. However, because of the limited number of observations and the high degrees of freedom, maximum lag length was set at three, and a three-lag structure was used.

For the multiple models used in the Toda–Yamamoto causality tests, the VIF values were checked to assess potential multicollinearity among the independent variables. In Model 5, the VIF value of *GINI* and *FPI* was 2.11, while it was 2.71 for *GDP_PPP* and *FPI* in Model 6 and 1.06 for *FI* and *FPI* in Model 9. In all of the models, the VIF values remained within the acceptable limits, which indicates that there was no multicollinearity among the independent variables.

The results in Table 3 show that *GINI* had a significant causal effect on both *FPI* and *FI* (Models 1 and 3). Similarly, *GDP_PPP* also had a significant effect on both *FPI* (Model 2) and *FI* (Model 4). In the multiple-variable analysis, *GINI* and *FPI* together had a significant causal effect on *FI* (Model 5). However, when examining the individual effects within the multiple-variable model, only *GINI*’s effect on *FI* was statistically significant (Model 5a, *p* < 0.05), while *FPI*’s effect was not (Model 5b, *p* = 0.0566). Similarly, while *GDP_PPP* and *FPI* jointly had a significant causal effect on *FI* (Model 6), neither variable had a statistically significant individual effect (Models 6a and 6b, *p* > 0.05), which indicates that the two variables had a joint effect. Additionally, Model 7 results confirmed that *FPI* had a significant causal effect on *FI* (*χ²* = 10.9157, *df* = 3, *p* = 0.0122 < 0.05). *FPI* had a strong effect on *HE* (Model 8), and *FI* had a significant causal effect on *HE* (Model 9). In the multiple-variable analysis, *FI* and *FPI* together had a significant causal effect on *HE* (Model 10); however, only *FPI* had a statistically significant individual effect, while *FI* did not (Model 10a: *p* < 0.01; Model 10b: *p* = 0.7532).

**Table 3:** Results of the Toda–Yamamoto causality test

X	Hypothesis		χ^2^	df	*p*	Result
1	*GINI* ⇏ *FPI* H_0_: No causality		25.2811	1	< 0.0001****	H_0_: Rejected
2	*GDP_PPP* ⇏ *FPI* H_0_: No causality		16.0365	4	0.003**	H_0_: Rejected
3	*GINI* ⇏ *FI* H_0_: No causality		17.8621	1	< 0.0001****	H_0_: Rejected
4	*GDP_PPP* ⇏ *FI* H_0_: No causality		38.5928	2	< 0.0001****	H_0_: Rejected
5	*GINI + FPI* ⇏ *FI* H_0_: No causality		23.4081	6	0.0003***	H_0_: Rejected
Submodel	5a	*GINI*_*sub*_ ⇏ *FI* H_0_: No causality	12.6706	6	0.0486*	H_0_: Rejected
	5b	*FPI*_*sub*_ ⇏ *FI* H_0_: No causality	12.2518	6	0.0566	H_0_: Not rejected
6	*GDP_PPP* + *FPI* ⇏ *FI* H_0_: No causality		22.2542	6	0.0011**	H_0_: Rejected
Submodel	6a	*GDP_PPP*_*sub*_ ⇏ *FI* H_0_: No causality	2.6631	6	0.8498	H_0_: Not rejected
	6b	*FPI*_*sub*_ ⇏ *FI* H_0_: No causality	6.6942	6	0.3501	H_0_: Not rejected
7	*FPI* ⇏ *FI* H_0_: No causality		10.9157	3	0.0122*	H_0_: Rejected
8	*FPI* ⇏ *HE* H_0_: No causality		13.1056	1	0.0003***	H_0_: Rejected
9	*FI* ⇏ *HE* H_0_: No causality		23.4081	5	0.0003***	H_0_: Rejected
10	*FPI* + *FI* ⇏ *HE* H_0_: No causality		16.2983	2	0.0003***	H_0_: Rejected
Submodel	10a	*FPI*_*sub*_ ⇏ *HE* H_0_: No causality	10.9157	2	0. 0122*	H_0_: Rejected
	10b	*FI*_*sub*_ ⇏ *HE* H_0_: No causality	3.4306	2	0.7532	H_0_: Not rejected

*p*-values were calculated according to the degrees of freedom

*p < 0.05

**p < 0.01

***p < 0.001

****p < 0.0001

The baseline model results show that all of the linear causality relationships were found to be significant. The model that jointly considers *GINI* and *FPI* showed a causal effect on *FI*. Similarly, the model that jointly considers *GDP_PPP* and *FPI* showed a causal effect on *FI*, and the model that jointly considers *FPI* and *FI* showed a causal effect on *HE*. Given that the multiple-variable models yielded statistically significant results and the direct effects were also statistically significant, we further assessed indirect causality using submodels. In this context, in the model that jointly considers *GINI* and *FPI* as causal variables (*p* < 0.0001), *GINI* had a significant individual effect on *FI* (*p* = 0.0486)*; however, contrary to our expectation of *FPI* being a mediator in the baseline model, we found that *FPI* did not have a statistically significant effect (*p* = 0.0566; borderline significance). Similarly, the model that jointly considers *GDP_PPP* and *FPI* was found to be significant (*p* = 0.0011); however, the individual effects of both variables were nonsignificant (*p* = 0.8498 for *GDP_PPP*, *p* = 0.3501 for *FPI*). Thus, we concluded that *FPI* did not have an indirect effect in the baseline model. In the model that jointly considers *FPI* and *FI*, the effect on *HE* was also found to be significant (*p* = 0.0001); however, while *FPI* had a statistically significant individual effect (*p* = 0.0019), *FI* did not (*p* = 0.2959). Based on these findings, we concluded that *FI* did not have a significant indirect causal effect in the baseline model.

Contrary to our expectations, in the baseline model, we found that *GINI* had an indirect effect on *FPI* in Model 5 and *FPI* had an indirect effect on *HE* in Model 10. To verify this and determine whether hypotheses H5a, H6a, and H10a provide indirect indications, we examine reverse causality by investigating the direct effect of *FPI* on *GINI* and *FI* on *FPI*. Accordingly, we propose the following new alternative hypotheses for the revised models:

- H_11_: *FPI* has a significant causal effect on *GINI*.
- H_12_: *FI* has a significant causal effect on the *FPI*.

Since the Toda–Yamamoto approach derives the optimal lag length and lag value primarily from the VAR model, and the maximum difference in stationarity (based on the ADF test results) yields the same outcome as forward causality, we proceeded with the model construction as follows.

$$\begin{aligned} \:{GINI}_{t}=&{\alpha\:}_{0}+\sum\:_{i=1}^{p}{\eta\:}_{1i}*{GINI}_{t-i}\\&+\sum\:_{j=p+1}^{p+dmax}{\alpha\:}_{2i}*{GINI}_{t-i}\\&+\sum\:_{i=1}^{p}{\varrho\:}_{1i}*{FPI}_{t-i}\\&+\sum\:_{j=p+1}^{p+dmax}{\beta\:}_{2j}*{FPI}_{t-j}+{\epsilon\:}_{2t} \end{aligned}$$

$$\begin{aligned} \:{FPI}_{t}=&{\gamma\:}_{0}+\sum\:_{i=1}^{p}{\eta\:}_{1i}*{FPI}_{t-i}\\&+\sum\:_{j=p+1}^{p+dmax}{\gamma\:}_{2i}*{FPI}_{t-i}\\&+\sum\:_{i=1}^{p}{\lambda\:}_{1i}*{FI}_{t-i}\\&+\sum\:_{j=p+1}^{p+dmax}{\zeta\:}_{2j}*{FI}_{t-j}+{{\Gamma\:}}_{2t} \end{aligned}$$

Table 4 presents the results of the causality test of FPI on GINI and FPI on FI. In Model 11, we found that FPI had a causal effect on GINI (*p* = 0.0048). However, in Model 12, FPI did not have a statistically significant effect on FI (*p* = 0.2674). These results indicate that food prices can influence income distribution, whereas food prices do not have a significant causal effect on food insecurity. Based on these linear findings, we can assume that FPI may have an indirect causal role on FI through GINI in the model that jointly includes FPI and GINI. However, in the model in which FI and FPI jointly influence HE, FPI did not have an indirect role, but only a significant individual effect within the multiple-variable model.

**Table 4:** Results of the causality test of *FPI* on *GINI* and *FI* on *FPI*

Model No	Hypothesis	χ^2^	df	*p*	Result
11	*FPI* ⇏ *GINI* H_0_: No causality	7.9501	1	0.0048*	H_0_: Rejected
12	*FI* ⇏ *FPI* H_0_: No causality	3.9456	3	0.2674	H_0_: Not rejected

**p < 0.01

**Fig. 3:**
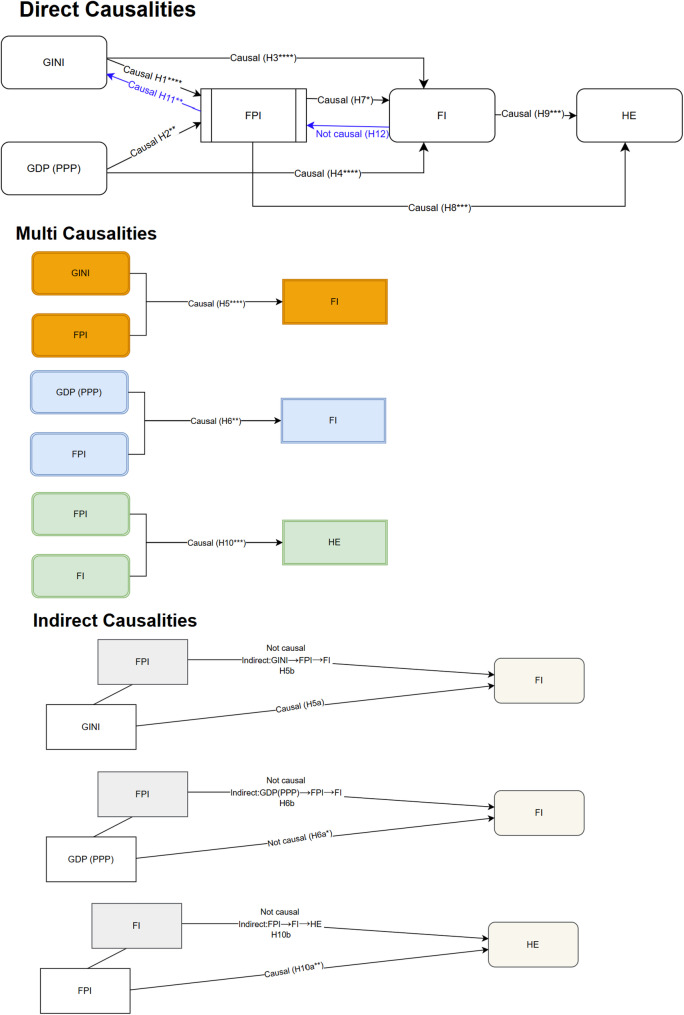
Causality results diagram

Figure 3 summarizes the causal pathways identified through the multicausality analysis. All single-variable models revealed statistically significant direct effects among the variables, confirming the robustness of the pairwise relationships. In the multivariable models, food insecurity (FI) was significantly affected when food price index (FPI) and income inequality (GINI) were included together as causal variables, supporting an indirect effect from FPI to FI through GINI with a 6-year lag. This finding confirms Hypothesis H5a and indicates that GINI mediates the relationship between FPI and FI. In contrast, while the joint inclusion of GDP_PPP and FPI also produced a significant effect on FI at the same lag length, the individual effects were not consistently significant when modeled separately, suggesting a synergistic interaction rather than a mediation mechanism. Similarly, in the model where FPI and FI jointly influenced health expenditure (HE), only FPI retained a significant direct effect at a 2-year lag. The absence of significance for FI in this combined model indicates that FI does not mediate the effect of FPI on HE. Thus, Hypothesis H12 was not supported. No significant indirect pathway from FPI to HE through FI was observed.

To identify low-income and higher-income countries, a K-means clustering analysis was conducted using GDP (PPP) and health expenditure (HE) values for the most recent year with complete data, 2022. The within-cluster sum of squares (WSS) and Silhouette scores were examined to determine the optimal number of clusters. The WSS value declined sharply from 96,476 at k = 1 to 31,107 at k = 2, after which the decrease became more gradual. Correspondingly, Silhouette scores were calculated as 0.657 for k = 2 and 0.688 for k = 3. Based on these results, a two-cluster solution (k = 2) was deemed the most appropriate for the classification.

**Fig. 4:**
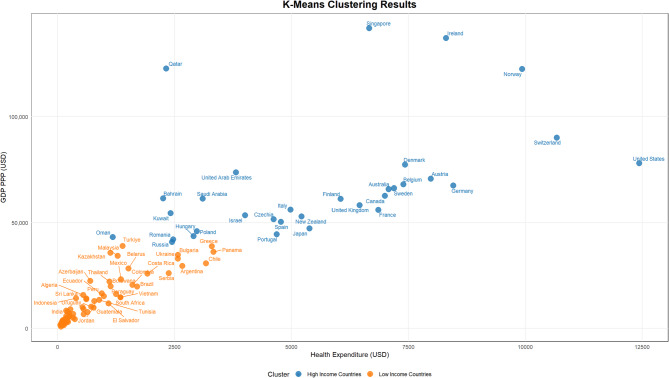
Clustering of countries by HE and GDP PPP (2022) to distinguish income groups

As a result of the clustering algorithm, countries were divided into two distinct groups. 32 countries with higher levels of GDP (PPP) and health expenditure were classified as “High-Income Countries,” while the remaining 67 countries with comparatively lower values for these indicators were categorized as “Low-Income Countries” (Fig. 4).

**Fig. 5:**
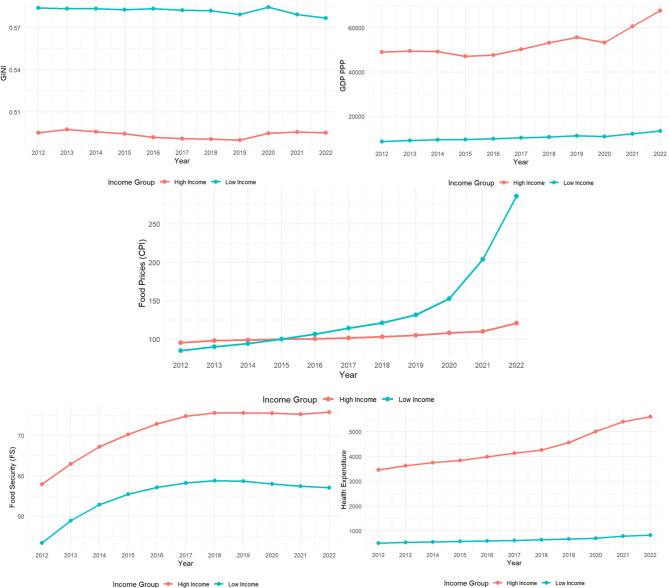
Indicators by income group (2012–2022)

To facilitate income-stratified analyses, the Prevalence of Severe Food Insecurity (FI) variable was substituted with the Global Food Security Index (FS), which offers an index-based, country-level measure of food security. This substitution was deemed methodologically appropriate for the causality-oriented analyses employed in this study, given that the primary objective was to assess directional relationships rather than estimate effect sizes. Furthermore, to enable the examination of food price dynamics across low- and high-income countries through time series models, the Consumer Price Index for Food (CPI) was utilized in place of the Food Price Index (FPI), as the latter is only available at the global level. The use of FS and CPI allowed for the implementation of subsequent time series analyses comparing food security and food price patterns across income groups. The relevant variable values for these income-stratified analyses are presented in Fig. 5.

According to Fig. 5, high-income countries exhibited a relatively stable increase in per capita GDP PPP and health expenditures, while the rise in food prices remained quite limited. In the same period, low-income countries also experienced growth in GDP PPP and health expenditures; however, the magnitude of this growth was considerably lower compared to high-income countries. The most striking difference is the sharp and rapid increase in food prices observed in low-income countries after 2019, whereas food prices in high-income countries rose much more modestly and at a slower pace. Furthermore, income inequality remained substantially higher in low-income countries, while it stayed lower and more stable in high-income countries. The food security (FS) indicator showed a marked upward trend in low-income countries, whereas in high-income countries, it remained relatively higher but stable. In terms of health expenditures, particularly after 2018, high-income countries displayed an accelerated growth rate, while low-income countries exhibited a more modest and stable increase. Supporting these trends, between 2012 and 2022, average per capita GDP PPP increased from 49,074 to 67,886 USD in high-income countries, and from 8,534 to 13,318 USD in low-income countries. Meanwhile, average health expenditure rose from 3,464 to 5,606 USD in high-income countries and from 509 to 826 USD in low-income countries. The Consumer Price Index– Food increased modestly from 95.6 to 121.0 in high-income countries, whereas in low-income countries it surged dramatically from 85.1 to 286.0. Over the same period, the FS indicator improved from 57.9 to 75.8 in high-income countries and from 43.4 to 57.1 in low-income countries, while GINI coefficients remained relatively stable.

In Table 5, the model numbers used to test hypotheses by income group correspond to those presented in Tables 3 and 4, with the only distinction being the substitution of the FPI variable with the CPI and the FI variable with the FS. Due to the limited observation period (2012–2022), and in contrast to the earlier time series models, degrees of freedom were constrained in cases where the maximum differencing order determined by the Akaike Information Criterion (AIC) required higher values. Furthermore, given the presence of trend-related issues in shorter series during the ADF tests, the more flexible KPSS test was employed as a complementary assessment of stationarity. Based on these results, all models—except for Model 9 in the high-income group—were evaluated using a maximum differencing order of 2 and a lag length of 1. For Model 9 in the high-income group, earlier stationarity allowed estimation with a lag length of 2.

**Table 5:** Comparison of Toda-Yamamoto causality results across income groups

	Low Income			High Income			Comparison with Wald	
Model No	χ ^2^	df	*p*	χ^2^	df	*p*	z	p
1	3.2115	1	0.0731	0.1833	1	0.6686	2.1413	0.0323*
2	9.1294	1	0.0025**	22.4414	1	< 0.0001****	−9.4130	< 0.0001****
3	1.1229	1	0.2893	725.1124	1	< 0.0001****	−511.9379	< 0.0001****
4	1.1004	1	0.2942	0.0211	1	0.8844	0.7632	0.4454
5	0.6255	2	0.7314	2.9402	2	0.2299	−1.1574	0.2471
*5a*	4.4538	2	0.1079	0.1934	2	0.9078	2.1302	0.0332*
*5b*	774.6794	2	< 0.0001****	11.2698	2	0.0036**	381.7048	< 0.0001****
6	34.1995	2	< 0.0001****	3.1823	2	0.2037	15.5086	< 0.0001****
*6a*	18.6440	2	0.0001***	19.8407	2	< 0.0001****	−0.5983	0.5496
*6b*	4659.7054	2	< 0.0001****	478.0004	2	< 0.0001****	2090.8525	< 0.0001****
7	186.8594	1	< 0.0001****	10.8759	1	0.0010**	124.4391	< 0.0001****
8	0.6613	1	0.4161	5.3858	1	0.0203*	−3.3407	0.0008***
9	26.2090	1	< 0.0001****	19.1492	2	0.0001***	4.9920	< 0.0001****
10	37.6464	2	< 0.0001****	97.9123	2	< 0.0001****	−30.1330	< 0.0001****
*10a*	228.2659	2	< 0.0001****	83.0643	2	< 0.0001****	72.6008	< 0.0001****
*10b*	117.2829	2	< 0.0001****	89.0944	2	< 0.0001****	14.0943	< 0.0001****
11	2.3551	1	0.1249	0.0493	1	0.8242	1.6304	0.1030
12	0.0560	1	0.8129	5.6874	1	0.0171*	−3.9820	0.0001***

*<0.051

**<0.01

***<0.00

****<0.0001

The results of the causality tests revealed that in Model 1 (GINI → CPI), no significant causality was found in either income group, but the Wald test indicated a significant group difference, with Low Income countries exhibiting a relatively stronger relationship (*p* = 0.0323). In Model 2 (GDP_PPP → CPI), both groups showed significant causality (*p* < 0.0001), with High Income countries demonstrating a stronger effect. For Model 3 (GINI → FS), the effect was only significant in High Income countries (*p* < 0.0001), indicating a stronger influence in this group (*p* < 0.0001). Model 4 (GDP_PPP → FS) showed no significant effects in either group. In Model 5 (GINI + CPI → FS), although the sub-components showed individual significant effects in the combined model with lag structures, the joint effect was not significant in both groups, and there were no differences between income groups. Similarly, in Model 6 (GDP_PPP + CPI → FS), while the sub-components exhibited individual significance within the combined model’s lag framework, the joint effect was significant exclusively in Low Income countries (*p* < 0.0001), revealing income group differences. When examined separately in Model 6b (CPI → FS), both income groups showed significant effects, though the magnitude was greater in Low Income countries. In Model 7 (CPI → FS), the effect was significant in both groups but stronger in Low Income countries. In Model 8 (CPI → HE), only High Income countries exhibited significant causality (*p* = 0.0203). In Model 9 (FS → HE), the relationship was significant in both groups but with varying temporal patterns and magnitudes: Low Income countries exhibited a stronger effect at a 1-year lag (*p* < 0.0001), while High Income countries showed a weaker effect at a 2-year lag. Model 10 (CPI + FS → HE) indicated significant effects in both groups, with High Income countries showing a stronger combined influence. In submodels 10a (CPI → HE) and 10b (FS → HE), both pathways were significant across groups, with High Income countries again demonstrating stronger effects. For Model 11 (CPI → GINI), no significant causality was observed in either group. Lastly, in Model 12 (FS → CPI), significant causality was found only in High Income countries (*p* = 0.0171), with a stronger effect compared to Low Income (*p* = 0.0001) (Table 5).

According to the significant findings presented in Table 5, the only mediating model was identified in high-income countries, highlighting the relationship between the Consumer Price Index, food security, and health expenditure. In high-income countries, a notable partial mediation mechanism was observed in the effect of the Consumer Price Index on health expenditure. Food prices directly influence health expenditure (CPI → HE), while food security also serves as an indirect pathway (CPI → FS → HE). The significant effects of both variables in the combined model (CPI + FS → HE), along with the strong relationships observed in the submodels, confirm the mediating role of food security in this process.

## Discussion

This study used a multicausality framework to investigate the direct and mediated relationships among income distribution, real income, food prices, food insecurity, and health expenditure. Utilizing the Toda–Yamamoto VAR approach, we revealed causal links even in nonstationary series, thereby overcoming the limitations of the traditional Granger causality analysis. Our findings showed that income distribution and real income have a marked influence on food prices and food insecurity, which affects public health expenditure. The extensive global dataset used in this study ensures that the results are robust and generalizable and highlight the complex interconnections among economic inequality, food security, and health financing. By delineating direct and indirect effects, our study offers nuanced insights that can guide integrated policy interventions. However, as the study relies on global aggregates, it may mask regional disparities. The interplay between food prices and income inequality might differ substantially across income groups or geographic regions. These results contribute to the literature by illuminating how multiple economic variables interact to influence social outcomes, thereby establishing a foundation for future research and policy reform. Our findings demonstrate that the Gini coefficient has direct and indirect effects on food prices and food insecurity. Specifically, high income inequality is associated with increased food prices and a concomitant deterioration in food security, as evidenced by the significant causal pathway found in this study. Moreover, the indirect influence of income distribution becomes more pronounced when it is examined in a multicausality framework; for instance, the synergistic interaction between the GINI coefficient and food prices further aggravates food insecurity. In parallel, real income also has a significant causal effect on food prices and food insecurity. Also, the identified lag lengths (e.g., 2-year and 6-year effects) are important because real-world policy outcomes often take time to emerge. For instance, health expenditure responses to food price shocks may occur after two years, reflecting policy reaction cycles and delayed health system impacts. Likewise, the 6-year lag linking food prices to food insecurity through inequality suggests deeper structural consequences. These lagged effects align with policy planning and evaluation periods, emphasizing the relevance of both immediate and long-term interventions.

To clarify the logic behind our mediation modeling, we provide two contrasting examples based on our findings. First, we observed that the Food Price Index (FPI) influences food insecurity indirectly through its effect on income inequality (GINI). This suggests that rising food prices may exacerbate income inequality—by increasing the burden on low-income households or shifting consumption patterns toward cheaper, less nutritious foods—which in turn leads to higher levels of food insecurity. In this case, the indirect path FPI → GINI → FI is supported by significant causal links in both the direct (FPI → GINI, GINI → FI) and multiple-variable models, and GINI retains significance in the submodel, indicating a partial mediation effect. In contrast, FPI was found to have a strong direct causal effect on health expenditure, but FI did not serve as a significant mediator in this relationship. One plausible explanation is that food price shocks may directly impact health costs—by increasing nutritional deficiencies or chronic disease incidence—without necessarily passing through food insecurity as an intermediary. This finding highlights that indirect causality requires a specific statistical structure: the independent variable must significantly cause both the mediator and the outcome, and the mediator must retain significance when modeled jointly with the independent variable. If only the mediator is significant in the combined model, it suggests full mediation, whereas significance for both implies partial mediation. To make these dynamics more intuitive, we added a visual representation (Fig. 3) illustrating the direct and indirect causal paths among FPI, GINI, FI, and HE.

The findings indicate that the complex interrelations among food prices, income inequality, GDP PPP, food insecurity, and health expenditure differ significantly across income groups. Although no statistically significant causal effect was found between income inequality and food prices in either group, the association appeared stronger in low-income countries—likely due to the heightened sensitivity of essential goods to income disparities. In contrast, GDP PPP had a significant causal effect on food prices in both groups, with a stronger impact observed in high-income countries, consistent with the demand-driven nature of inflation in advanced economies.

Income inequality significantly influenced food insecurity only in high-income countries, where socio-economic disparities more clearly shape food access. In contrast, in low-income countries—where food insecurity is mostly structural—changes in inequality tended to maintain rather than worsen existing conditions. Notably, GDP PPP showed no significant relationship with food insecurity in either group, suggesting that economic growth alone may not be enough to improve food access. While the combined effect of inequality and food prices was not significant, the individual impact of food prices on food insecurity was especially strong in low-income countries, emphasizing their increased vulnerability to food inflation without strong social protection systems. These findings align with Ginn [34], who, using a panel local projections approach across 145 countries, demonstrated that income growth significantly reduces food insecurity only in low-income settings, where food access depends more directly on household income. In high-income countries, however, food insecurity seems less responsive to economic growth, likely due to structural protections and more stable consumption patterns. This explains our observation that GDP PPP had no direct effect on food insecurity in either group, despite its strong effect on food prices. Additionally, the significant relationship between income inequality and food insecurity in high-income countries further supports the idea that socio-economic stratification plays a larger role in shaping food access where structural barriers are less severe.

The combined impact of purchasing power-adjusted GDP and inflation was found to trigger food insecurity only in low-income countries, highlighting how poorer economies remain vulnerable to macroeconomic instability. Evidence from Brazil shows that food insecurity is strongly linked to poorer child health and nutrition outcomes and partly explains the health gap between income groups [35]. These findings highlight how, in lower-income settings, macroeconomic shocks can lead to immediate household-level nutritional deprivation, especially for vulnerable populations like children. While citizens in high-income countries are protected by various safety nets, those in low-income settings face the same economic challenges without comparable buffers, often paying the cost directly at the household level through reduced access to food.

Our findings demonstrate a nuanced relationship between food insecurity and health expenditure across income groups. These relationships manifested as a stronger and more immediate impact in low-income countries, whereas in high-income countries, the effect was weaker and appeared over a longer time horizon. This likely reflects the rapid health deterioration associated with undernutrition in contexts with limited healthcare infrastructure [36].

Herman et al. [37] showed that in high-income contexts, improvements in diet quality led to significant health gains and cost savings, though these benefits accumulate gradually over time. Their results reinforce our finding that, in high-income countries, the health expenditure impact of food insecurity develops more slowly due to the delayed onset of nutrition-related conditions.

This interpretation is further supported by a study conducted by Ginn [38], which demonstrates that healthcare expenditure is more income-elastic in low-income countries, particularly during non-expansionary economic phases. These findings reveal that health spending is more sensitive to macroeconomic shocks, exposing underlying vulnerabilities in such contexts. In contrast, high-income countries tend to maintain more stable healthcare spending patterns, even during downturns, reflecting greater system resilience and institutional protection.

Furthermore, we found that the combined impact of food insecurity and food inflation significantly raises health expenditures, especially in high-income countries. This may demonstrate how food crises affect health systems in developed economies through indirect but systematic pathways by reducing diet quality and increasing healthcare demand. Choe and Pak [39] found that food insecurity distinctly increased healthcare utilization in Korea. This supports the idea that, in developed settings, food insecurity imposes an indirect yet structured burden on health systems.

Our findings did not reveal a causal effect of food prices on income inequality in either income group, highlighting the limited short-term ability of price shocks to alter societal income distribution. In contrast, food insecurity was found to significantly influence food prices only in high-income countries, indicating a feedback loop where demand-side shocks increase market volatility. This asymmetry suggests that food crises cause more complex price dynamics in advanced economies, while these mechanisms are more limited or absent in low-income settings. However, existing research emphasizes that shifts in income inequality can greatly impact food prices by affecting consumer demand and the structure of the food market. Blok et al. showed that changes in income distribution influence food prices, with high-income groups demanding more healthy foods than low-income groups. They also suggested that lowering the cost of nutritious foods is a long-term strategy to reduce inequalities [40]. In many developing countries, high economic growth can be linked to decreased food insecurity. However, the effect of economic growth on food insecurity is directly tied to income fluctuations. Economic growth combined with income inequality places more pressure on low-income groups than on high-income groups [41]. In this context, the link between income distribution, food prices, and food insecurity is vital for discussions on public health policies. Income inequality and fluctuations in food prices worsen food insecurity for low-income populations. Research shows that food insecurity is more common in societies with high income inequality [42].

The multivariate analysis revealed that when considering the Gini coefficient and food prices together, GINI had a statistically significant individual effect on food insecurity. In contrast, food prices alone had a borderline significant effect. High food prices can lead to food insecurity by limiting low-income individuals’ access to adequate and balanced nutrition [43]. Gregory and Coleman-Jensen found that a $10 increase in food prices results in a 2.5% rise in household food insecurity, and that low-income households living in high-cost food regions face a greater risk of food insecurity [7]. The stronger causal link among income inequality, food prices, and food insecurity underscores the need for an integrated approach to reduce food insecurity risk.

Our results indicate that, in multivariate analyses, the impact of real income on food insecurity becomes more pronounced when combined with food prices. The reinforcing effect of income level on food insecurity suggests that low-income households find it increasingly difficult to access food during economic downturns, with long-term impacts. This emphasizes that income level is both a short- and long-term factor influencing food insecurity.

The literature on the macroeconomic determinants of food insecurity has shown that low household income, unemployment, and the level of economic development are key drivers of food insecurity [44, 45]. The FAO’s 2020 report states that although an increase in per capita income reduces food insecurity in the short term, it does not have the same impact on long-term indicators, such as child malnutrition [41]. Similarly, the International Monetary Fund’s 2024 report states that high food inflation threatens food security, particularly for low-income households, and policies aimed solely at reducing food prices are insufficient to mitigate food insecurity [46]. Given that per capita income affects both food prices and food insecurity, addressing food insecurity effectively requires integrated policies that stabilize food prices and increase the income level of low-income households. Therefore, holistic strategies that balance food price regulations and income-enhancing policies should be implemented in the fight against food insecurity.

In this study, *FPI* was revealed to have a robust and direct effect on both *FI* and *HE*. This finding indicates that fluctuations in food prices are a key driver of food insecurity, with higher food prices leading to increased levels of food insecurity. Concurrently, the strong direct relationship seen between *FPI* and *HE* underscores the substantial economic burden imposed by food price volatility on public health systems. When *FPI* was evaluated together with *GINI* and *GDP_PPP*, we found a synergistic effect; the combined effect of these variables exacerbates the adverse impact on food security and public health expenditure, which highlights the interconnected nature of these economic and social indicators. Notably, relationship between food price and health expenditure showed important variation across income groups: the effect of food prices on health expenditure was statistically significant only in high-income countries. In these settings, food inflation may constrain household budgets more sharply, forcing shifts in spending priorities and contributing to increased medical expenses. In contrast, in low-income countries expenditures remained relatively stable and appeared less sensitive to price shocks.

The continuous rise in food prices globally is a major challenge today. Since 2020, global crises have caused considerable disruptions in agricultural production and supply chains, leading to high food prices, and, in turn, directly affecting household incomes and restricting access to essential food resources [4, 47–49]. Recent evidence from the Caribbean context highlights how surging imported food prices significantly eroded consumer welfare during global shocks, including the COVID-19 pandemic, emphasizing the broader vulnerability of import-dependent regions to global food price volatility [50]. Additionally, Forgenie et al. [51] showed that net food-importing developing countries exhibit substantial price elasticities across key food groups, underscoring the critical role of income and price sensitivities in shaping consumption behavior and food security outcomes. Kedir et al. [47] found that since the start of the Russia–Ukraine war, grain price increases have resulted in increased malnutrition rates in low-income countries across Africa and Asia. The impact of food prices on food insecurity is manifested directly through reduced purchasing power and indirectly through broader economic mechanisms that influence household food access and affordability.

In addition to its direct role, food insecurity has a long-term effect on health expenditure. However, in our models, when food insecurity was analyzed alongside food prices, we found that it had a comparatively limited individual mediating role. While food insecurity may contribute to increased health expenditure in the long run, our findings suggest that food prices exert a more dominant and statistically significant influence on health spending. Nevertheless, the relationship between food insecurity and health expenditure may vary considerably across economic contexts—particularly in low-income countries. In such settings, food insecurity may paradoxically lead to lower observed healthcare expenditures, as financial constraints hinder access to necessary medical services, thereby obscuring the true burden of unmet health needs despite worsening health outcomes. Consequently, expenditure-based health indicators may underestimate the actual health impact of food insecurity in these contexts. Indeed, empirical findings from the literature further emphasize the complex and context-dependent nature of this relationship. For instance, a study conducted in Sub-Saharan Africa indicated that food insecurity significantly increased the risk of chronic conditions such as cardiovascular diseases, diabetes, and hypertension, thus elevating long-term healthcare demands despite potentially lower immediate expenditures due to restricted access [52]. Similarly, another investigation in Sub-Saharan African countries demonstrated that food insecurity was associated with poorer health outcomes and heightened vulnerability to chronic health conditions, further compounding long-term healthcare burdens [53]. Conversely, evidence from higher-income settings, such as the United States, reveals a bidirectional yet asymmetric relationship, where food insecurity substantially increases future healthcare expenditures (approximately 25% higher expenditures among food-insecure households), whereas higher healthcare spending only modestly increases subsequent food insecurity [12].

Furthermore, our VAR-based multicausality approach enabled the identification of distinct short- and long-term causal pathways. For instance, the short-term (2-year) lag between food price shocks and health expenditure likely reflects immediate financial strain on households, whereas the long-term (6-year) mediated impact of food prices on food insecurity through income inequality underscores deeper structural processes. Empirical evidence supports the notion that short-term effects predominantly reflect households’ immediate responses to sudden economic pressures. For example, Pybus et al. [54] highlight that food insecurity demands rapid budgetary adjustments, especially among low- and middle-income families in high inequality regions, leading to reduced food expenditure and compromised nutritional quality. Similarly, Neerland et al. [55] conducted an extensive evidence mapping review, which highlighted that structural factors such as systemic racism, housing instability, and lack of access to affordable healthcare significantly exacerbate food insecurity, especially among marginalized populations. Their analysis underscored that food insecurity is not merely the outcome of temporary economic shocks but is deeply embedded within long-term structural inequalities that persistently undermine health outcomes. These chronic conditions—rooted in entrenched social determinants—demand more than short-term economic relief and instead require comprehensive, equity-focused policy reforms to effectively reduce health burdens associated with food insecurity. Similar concerns have also been raised in country-specific analyses such as Nigeria, where population growth, inflation, and globalization exert quantile-dependent and often adverse effects on food production and security, highlighting the need for context-specific policy solutions [56]. This is corroborated by Elia et al. [57], who found that structural inequalities significantly influence long-term nutritional outcomes and chronic health conditions, further burdening health systems. Collectively, these findings underscore the necessity of comprehensive strategies addressing both immediate economic shocks and the underlying structural inequalities that perpetuate food insecurity and health system pressures.

Studies have shown that food insecurity is associated with increased hospital admission rates and healthcare expenditure [58, 59]. However, our findings underscore the importance of distinguishing between the direct effects of food price volatility and the mediating role of food insecurity within the broader socioeconomic landscape.

Food insecurity alone is not a dominant determinant of health expenditure, which suggests that economic, environmental, and sociodemographic factors contribute multidimensionally to healthcare costs [60–62]. However, our analysis reveals that the relationship between food prices, food insecurity, and health expenditure varies significantly by income level. In high-income countries, food inflation not only increases direct household medical spending but also undermines food security. This, in turn, raises healthcare costs through poorer diet quality and higher rates of chronic disease [63]. This two-step mechanism indicates that even with robust welfare systems, food price shocks can impose a measurable burden on advanced health systems. In contrast, this indirect pathway is not evident in low-income countries. In these nations, limited purchasing power, weak insurance coverage, and underfunded health services may prevent food insecurity from translating into recorded health expenditures. This could occur because care is forgone or subsidized.

These findings underscore the need for tailored policy responses. High-income countries may benefit from integrating food price stabilization and health promotion. Meanwhile, low-income countries must first focus on improving access to nutritious food and essential health services.

The COVID-19 pandemic led to a significant increase in food insecurity. According to the World Health Organization [3], 2.33 billion people worldwide faced food insecurity in 2023. The economic constraints on access to healthy foods have further intensified the pressure on food security [64]. Otero et al. [10] found that in countries with inequalities in food access, particularly among working-class families, financial constraints limit access to high-quality foods, increasing exposure to low-cost, energy-dense, and nutrient-poor food options. As demonstrated in our study, the strong causal relationship between food prices and healthcare expenditures is a challenge to individual and household nutritional choices and a significant burden on healthcare systems.

Limited access to nutritious foods worsens malnutrition and increases the prevalence of noncommunicable diseases (NCDs), such as diabetes, hypertension, and cardiovascular diseases, thereby significantly increasing the global burden of disease [65]. Between 2011 and 2030, NCDs are estimated to cost the global economy $30 trillion in terms of productivity losses and increased healthcare expenditures [66]. In countries with a state-funded healthcare system, the growing burden of chronic diseases is leading to the allocation of a greater proportion of public health budgets to preventable health conditions [67]. Although healthcare costs associated with NCDs can be managed through nutrition-based interventions, the lack of preventive health policies has shifted expenditures toward medical treatment rather than prevention [68, 69].

Berkowitz et al. [58] emphasized that in societies with high income inequality, price regulations and social support mechanisms to improve access to essential and nutritious foods are critical in reducing healthcare expenditure. To reduce long-term healthcare costs, the focus of health policies must be extended beyond disease treatment on broad-based economic and social policies that ensure food accessibility and lowered financial barriers to healthy nutrition [12].

Our findings also suggest that food prices have a significant effect on income inequality. While *FPI* was found to have a statistically significant causal effect on *GINI*, the data did not support a direct causal link between *FI* and *FPI*. This finding suggests that food prices may drive food insecurity indirectly through their effect on income inequality. The determinants of food pricing policies are heavily influenced by macrolevel market forces, such as supply–demand dynamics, food production, supply chains, and government regulations. Consequently, food insecurity has a relatively weak direct effect on food prices, as its impact is often overshadowed by economic and policy-driven factors [70]. Our findings are in line with those of Marson and Saccone, who showed that [71] sudden increases in food prices exacerbate income inequality in developed countries, with low-income populations being disproportionately affected by these fluctuations. The contribution of food price volatility to income inequality is mainly linked to low-income households allocating a substantial proportion of their total expenditure to food, making them highly vulnerable to a decline in purchasing power [72]. Similarly, Marktanner and Noiset [73] showed that doubling of food prices in low-income countries substantially increases poverty rates. Given that countries with high income inequality are often developing economies, it is crucial that food pricing policies shield vulnerable populations from the adverse effects of price fluctuations through targeted subsidies, market stabilization mechanisms, and social safety nets.

Methodologically, the use of a multicausality framework in this study, which integrates the Toda–Yamamoto approach and VAR, is a significant advancement over conventional single-equation models. This design accommodates the challenges of nonstationary time-series data and allows the disaggregation of joint and individual effects of the key variables. Consequently, the results of our study offers a richer, more holistic understanding of how income distribution, real income, food prices, and food insecurity collectively influence public health expenditure. This conceptual innovation contributes to the literature by demonstrating that incorporating direct and mediated effects provides a more comprehensive perspective on the complex interplay of macroeconomic factors.

Our findings highlight the key role of income distribution in shaping food security and public health expenditure. Reducing economic inequality through tax reforms, social transfer mechanisms, and investments in education can enhance food access while alleviating the financial pressure on healthcare systems. Equitable income distribution is key to promoting social welfare and sustainable health financing. Given the strong impact of food prices on food insecurity and health expenditure, stabilizing food markets should be a policy priority. Strategic food reserves, agricultural support programs, and international cooperation can help mitigate price volatility and its adverse effects. By cushioning the impact of price shocks, such measures can improve food availability and reduce the strain on public health resources. Additionally, increasing health expenditures, driven partly by food insecurity and price fluctuations, raise concerns about fiscal sustainability. It is essential to strengthen preventive health policies and primary healthcare systems to manage these costs. Proactive, early interventions can curb the increase in expenditure while ensuring the provision of high-quality and accessible healthcare services, making health systems more resilient in the face of economic fluctuations.

### Limitations

The limited data coverage and time span cannot capture long-term causal effects. Additionally, the study design does not allow for the use of models, such as VAR, autoregressive distributed lag, unrestricted error correction model regression, and restricted error correction model regression, which can capture more dynamic effects. This limitation makes it more challenging to determine the direction of causality. Specifically, variables, such as health expenditure and missing or limited yearly observations, require forecasting methods, which may lead to deviations from the actual data. The same situation applied to the comparison of low- and high-income countries, where the scarcity of observations was the major limitation. Furthermore, Model 5 could not be fully constructed despite a higher optimal lag length because the degrees of freedom in the multiple-variable model were high; thus, the maximum lag length had to be limited to three. This constraint limited the range suggested by the information criteria and the modeling of long-term relationships. It is also directly related to the number of observations, as each additional explanatory variable in a multiple causality model increases the number of parameters in the system, necessitating a higher number of observations. Another limitation arises from the differences in the definitions and methodologies used by different institutions, which partially restricts the direct comparability of different variables.

Although the Toda–Yamamoto method does not require the consideration of structural breaks and economic shocks, this may limit the model’s accuracy. For instance, fluctuations in food prices during the 2007–2008 global financial crisis, 2010–2011 grain price shocks, and the COVID-19 pandemic can be analyzed more effectively using advanced modeling techniques that provide more robust causal insights. However, such techniques require longitudinal structural models and a sufficient number of observations to ensure that the models function properly.

## Conclusion

The findings of this study demonstrate that macroeconomic variables, particularly income inequality and real income, directly influence food prices and that fluctuations in food prices significantly affect social outcomes, including food insecurity and health expenditure. Our multicausality framework further reveals that seemingly nonsignificant relationships in isolation may become significant when modeled jointly, indicating the presence of indirect or synergistic effects that are often masked in single-equation models. Specifically, food prices affect food insecurity indirectly via income inequality, and real income amplifies this mechanism. Similarly, food price shocks directly increase health expenditures, independent of food insecurity, in some models.

These findings suggest that policy responses should address not only individual determinants but also their interactions. Based on this, we propose the following targeted, evidence-based interventions:

- Reduce income inequality through progressive taxation, universal child benefits, and conditional cash transfer programs that incentivize school attendance and healthcare utilization among vulnerable populations.
- Implement food voucher systems or targeted food subsidy schemes, particularly in urban low-income areas, to offset the adverse effects of food price volatility.
- Stabilize food markets through national strategic food reserves, minimum price guarantees for essential commodities, and regional food security pacts that reduce dependency on volatile global markets.
- Strengthen preventive health strategies, such as early nutrition screening programs, maternal and child health education, and community-based dietary interventions, to reduce healthcare burdens caused by malnutrition-related diseases.
- Expand agricultural support for smallholder farmers through input subsidies and infrastructure investment, thereby increasing domestic food supply resilience and reducing exposure to import shocks.

Our results highlight the heterogeneity in how food inflation and food insecurity influence health expenditure across different economic contexts. Rather than acting as a universal driver of healthcare costs, food insecurity interacts with broader structural factors—such as national income levels, welfare systems, and healthcare accessibility—to produce divergent outcomes. In wealthier nations, the capacity to document and respond to food-related health stressors reveals a dual burden, whereby rising food prices not only impact dietary choices but also translate into increased medical spending. Conversely, in low-income settings, systemic limitations may suppress visible links between food inflation and health expenditure, not due to a lesser impact, but because care is often unaffordable or inaccessible.

These findings emphasize the importance of context-sensitive strategies in public health and economic policymaking. While advanced economies must address the health consequences of volatile food prices through integrated food and healthcare policy frameworks, the priority in lower-income countries lies in expanding the foundational access to a nutritious diet and essential health services.

Future research should further explore the mechanisms through which economic shocks shape health outcomes across income groups, especially in settings where expenditures may not fully capture unmet health needs. Additionally, regional differences—such as those observed between Sub-Saharan Africa and Southeast Asia—necessitate context-specific strategies.

Lastly, as health expenditures continue to rise globally, their link to food-related economic pressures must not be ignored. Strengthening primary healthcare systems, promoting nutrition education, and integrating social determinants into national health planning are essential for maintaining fiscal sustainability. Future models should also incorporate variables such as unemployment, female labor force participation, and environmental shocks, which likely mediate the complex dynamics between food insecurity and health outcomes.

## Supplementary Information

Supplementary Material 1.

## Data Availability

The data utilized in this study are openly accessible at the following link: https://zenodo.org/records/15736704 (accessed on June 25, 2025).

## Abbreviations

ADF: Augmented Dickey–Fuller
AIC: Akaike information criterion
CPI: Consumer price index– food
CUSUM: Cumulative sum (test for structural stability)
CUSUMSQ: Cumulative sum of squares (variance stability test)
FI: Prevalence of severe food insecurity
FIES: Food insecurity experience scale
FPI: Food price index
FS: Global food security index
GDP_PPP: Gross domestic product per capita (purchasing power parity)
GINI: Gini coefficient (income inequality indicator)
HE: Health expenditure
KPSS: Kwiatkowski–Phillips–Schmidt–Shin (stationarity test)
MAPE: Mean absolute percentage error
NCDs: Noncommunicable diseases
RMSE: Root mean square error
SOFI: State of food security and nutrition in the world
VAR: Vector autoregression
VIF: Variance inflation factor
WID: World inequality database
WSS: Within-cluster sum of squares
