# Supplementary material for: Examining the interconnections among income, food prices, food insecurity, and health expenditure: a multicausality approach
Source: BMC Public Health. 2025 Aug 14;25:2778. doi: 10.1186/s12889-025-24153-6 (PMC12351902; doi:10.1186/s12889-025-24153-6)
Supplement: Supplementary file 1 — Supplementary Material 1. [file 12889_2025_24153_MOESM1_ESM.docx]

Supplementary Appendix A: Bai Perron Tests’, CUSUM and CUSUM SQ Tests’ Graphics

| 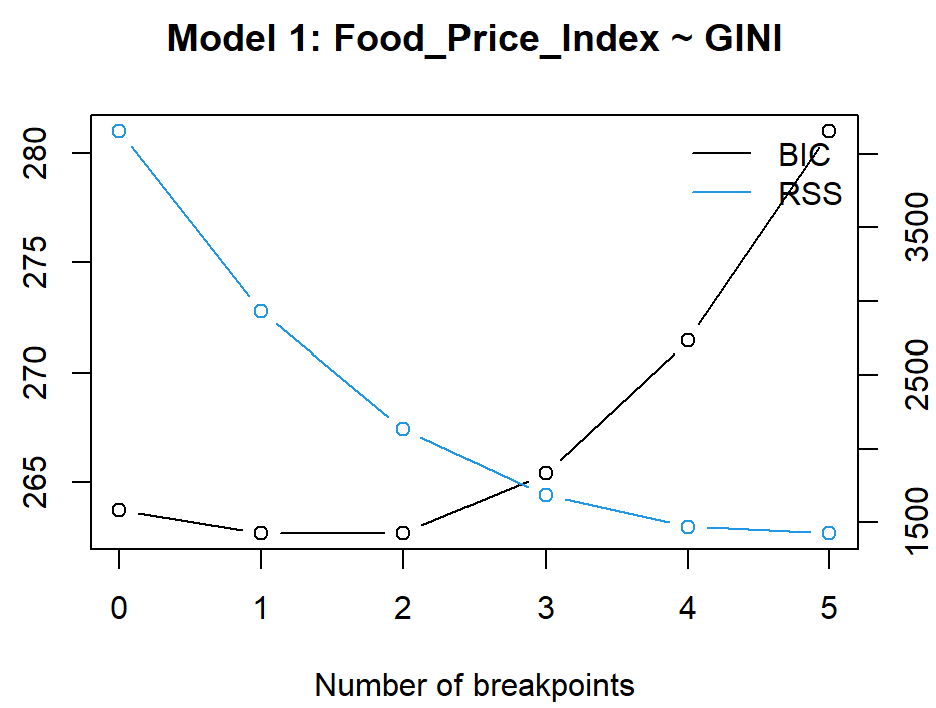 | | 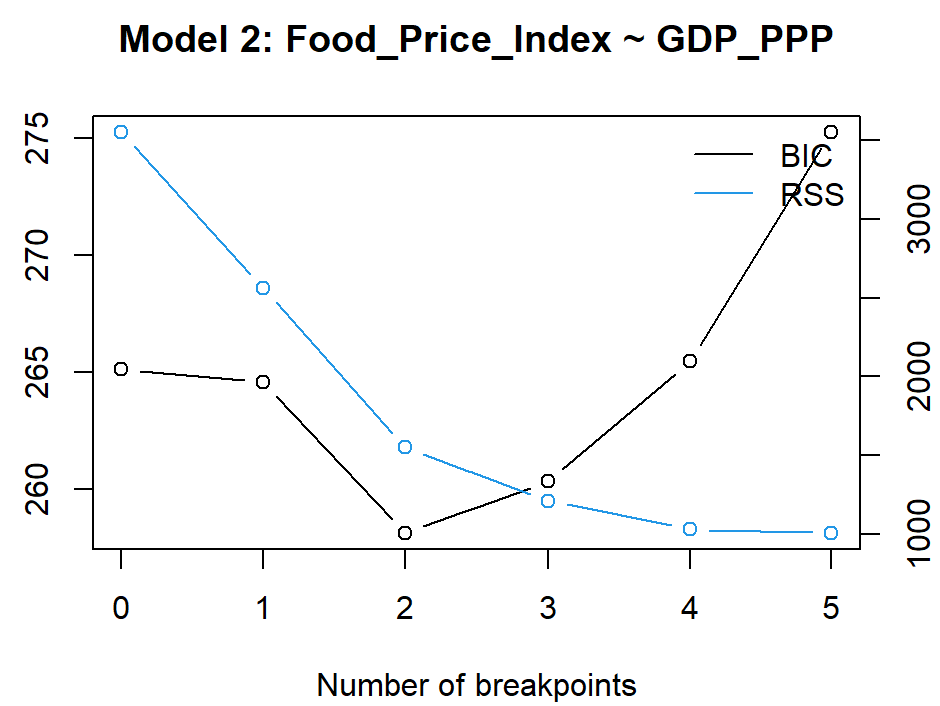 | |
| --- | --- | --- | --- |
| 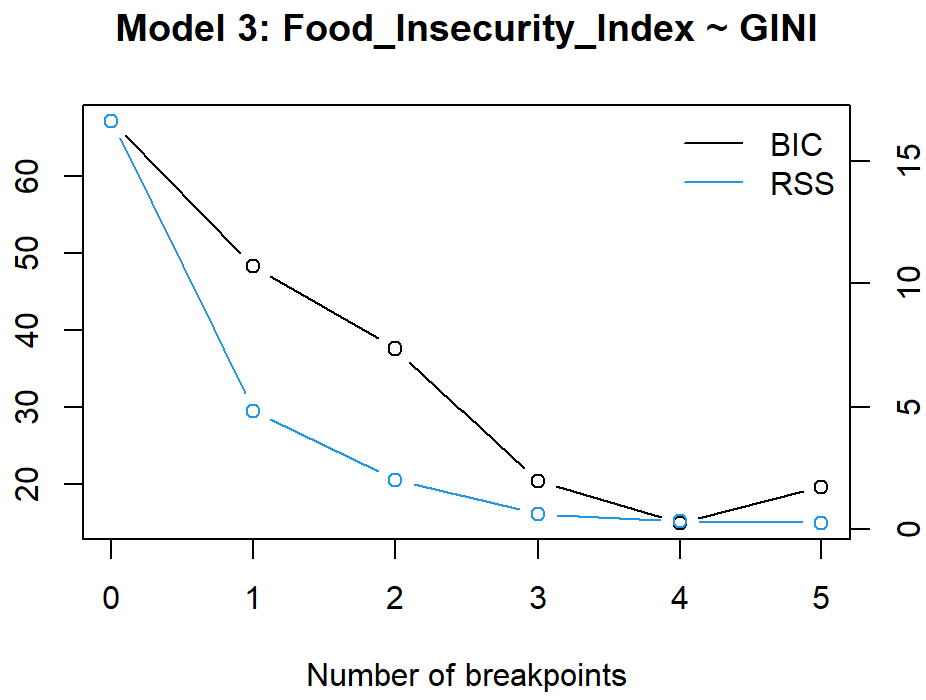 | | 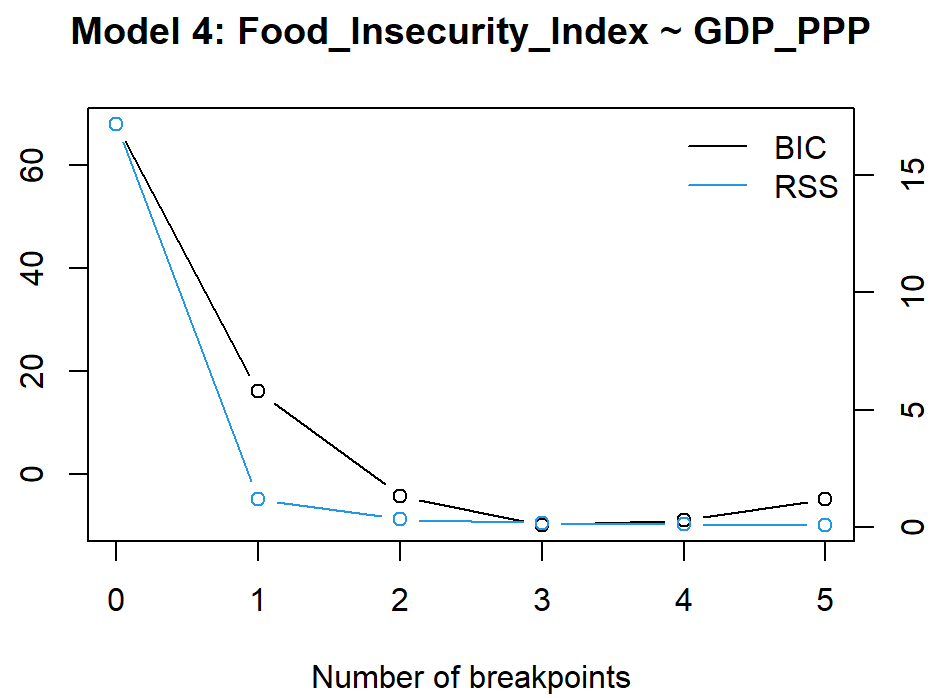 | |
| 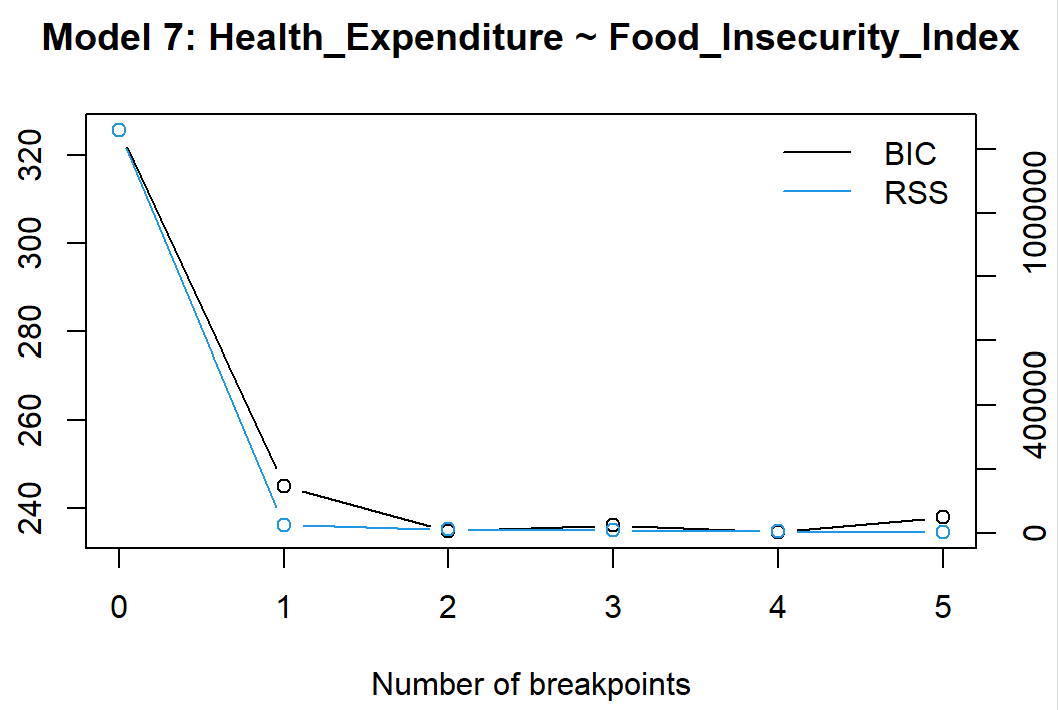 | | 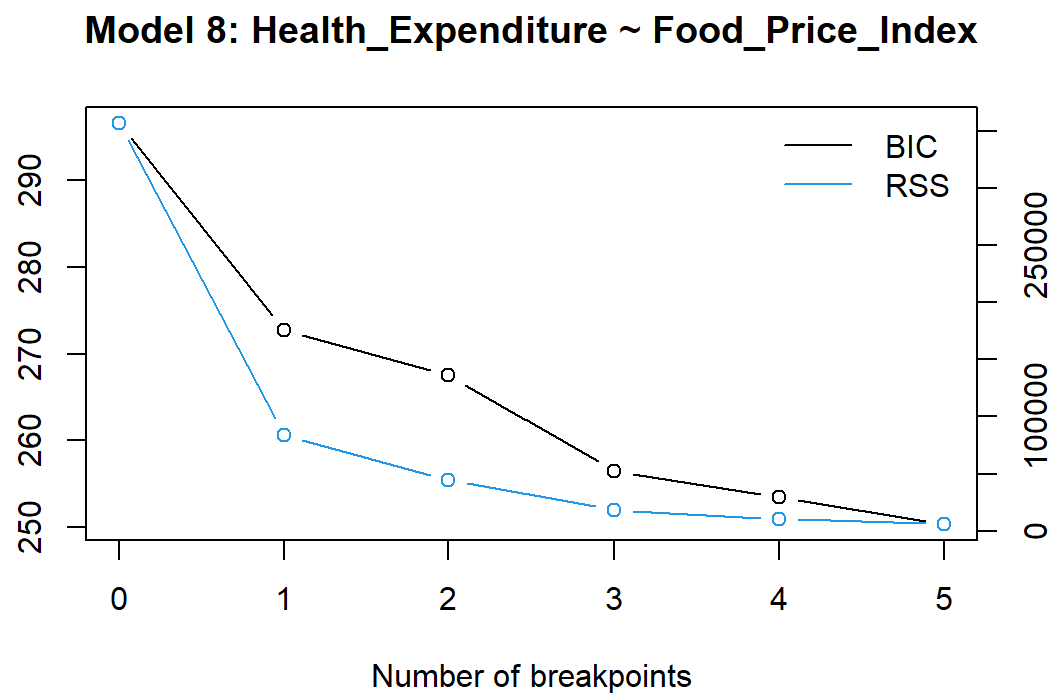 | |
| 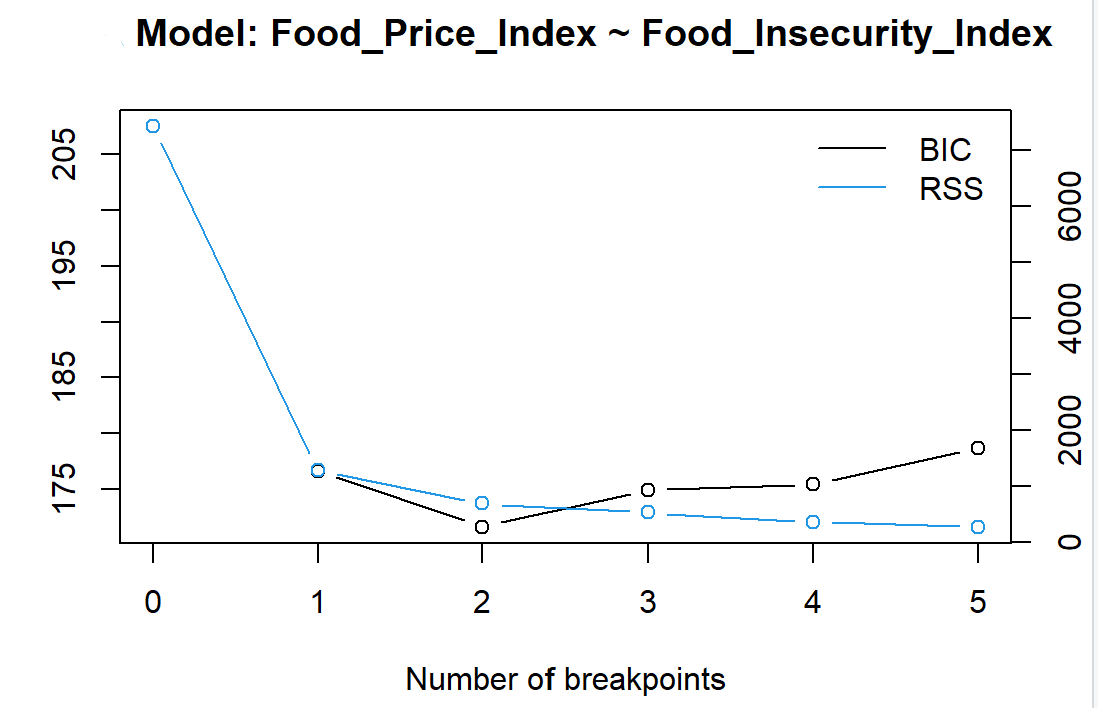 | | | |
| 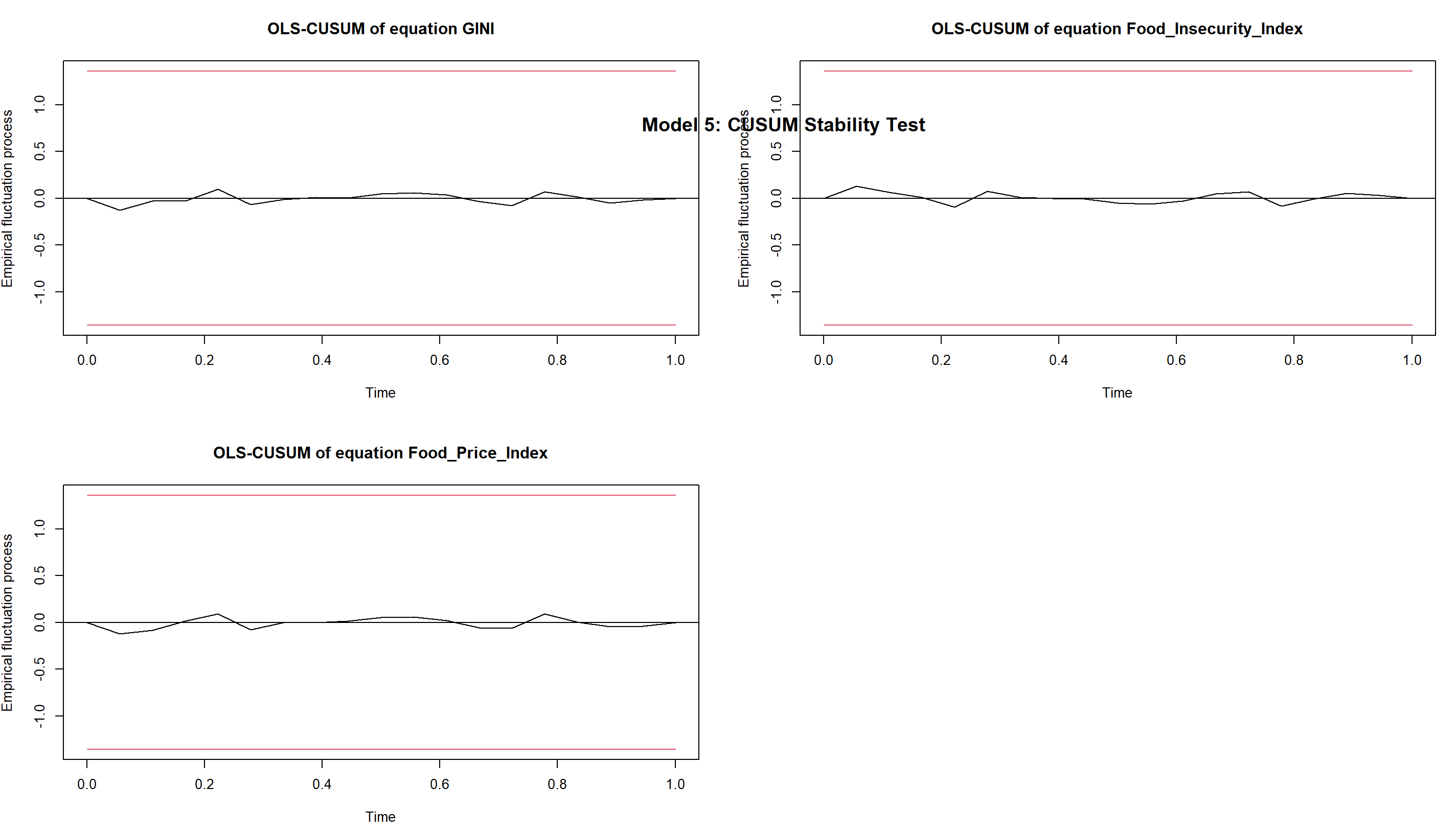 | | | 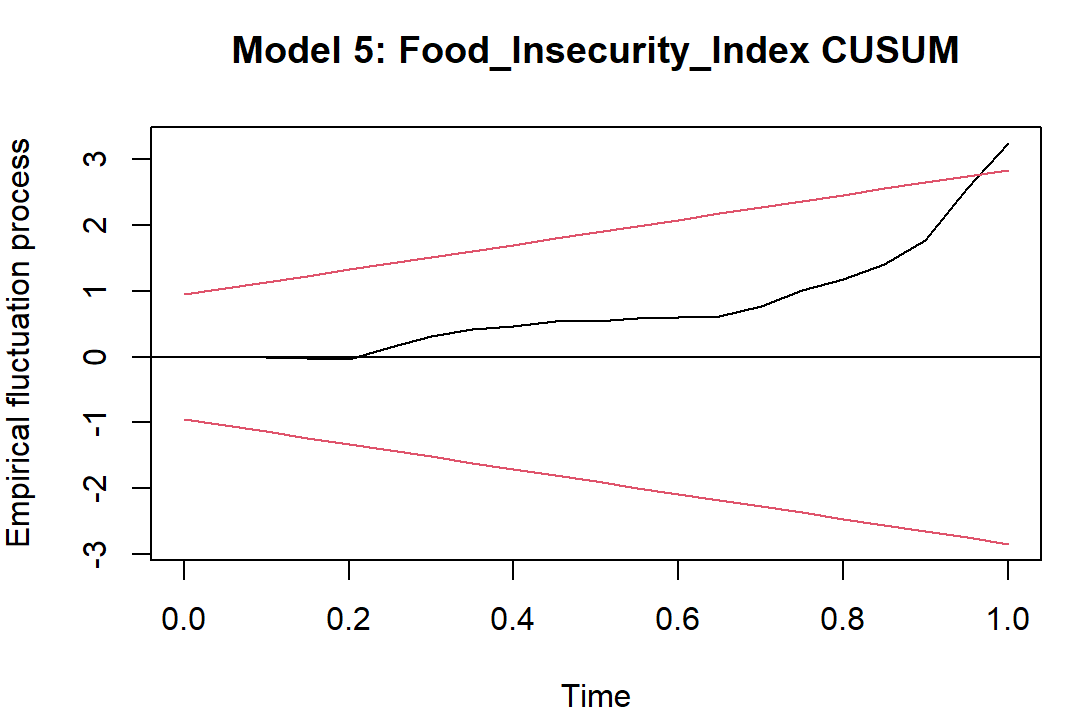 |
| 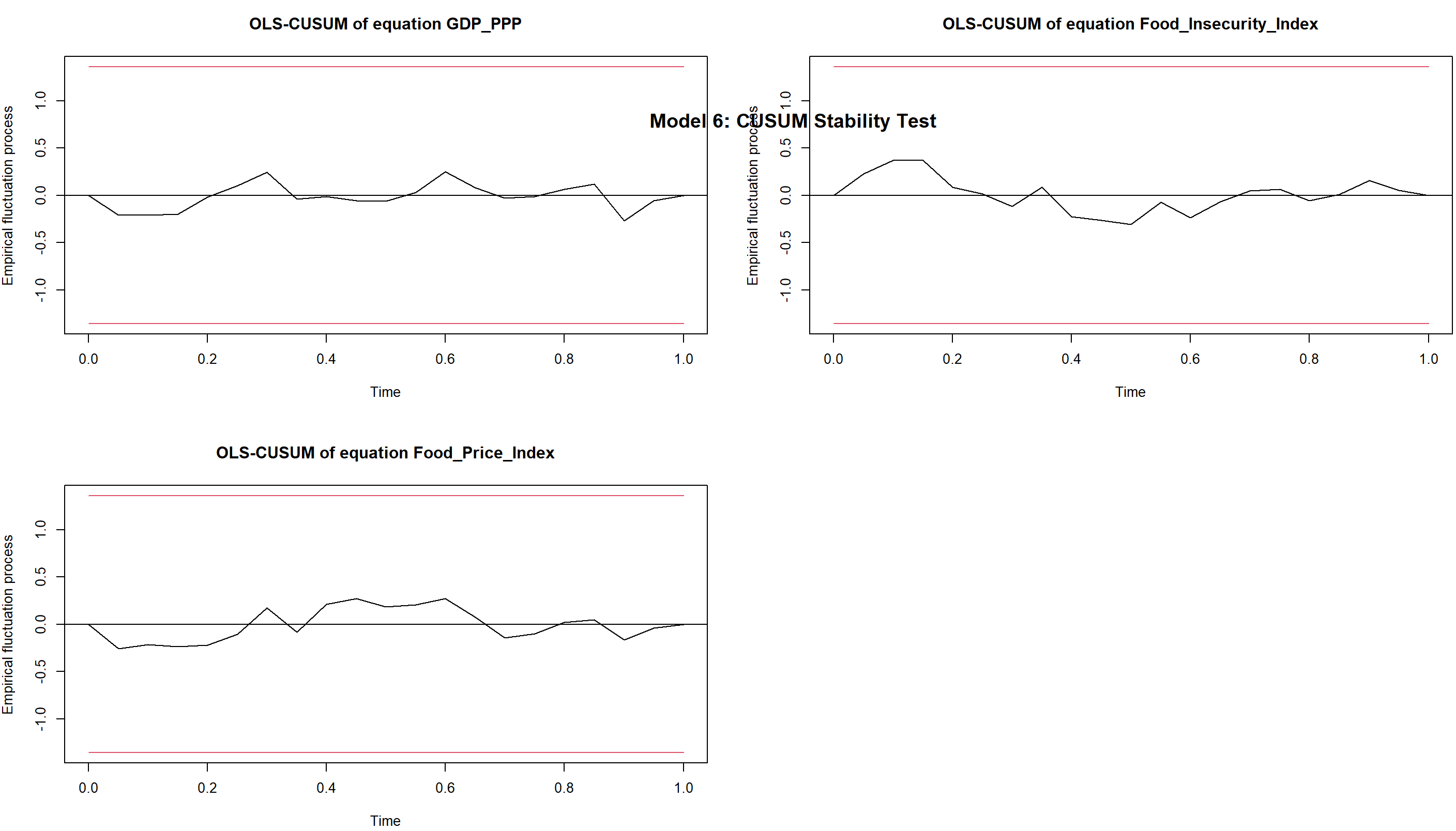 | | | 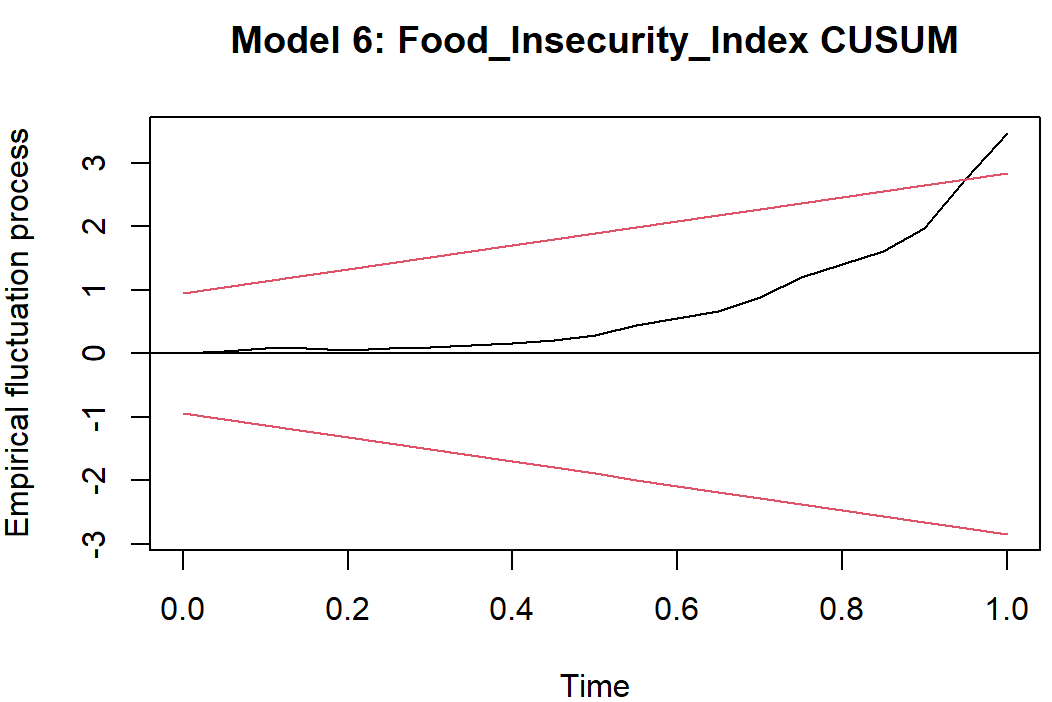 |
| 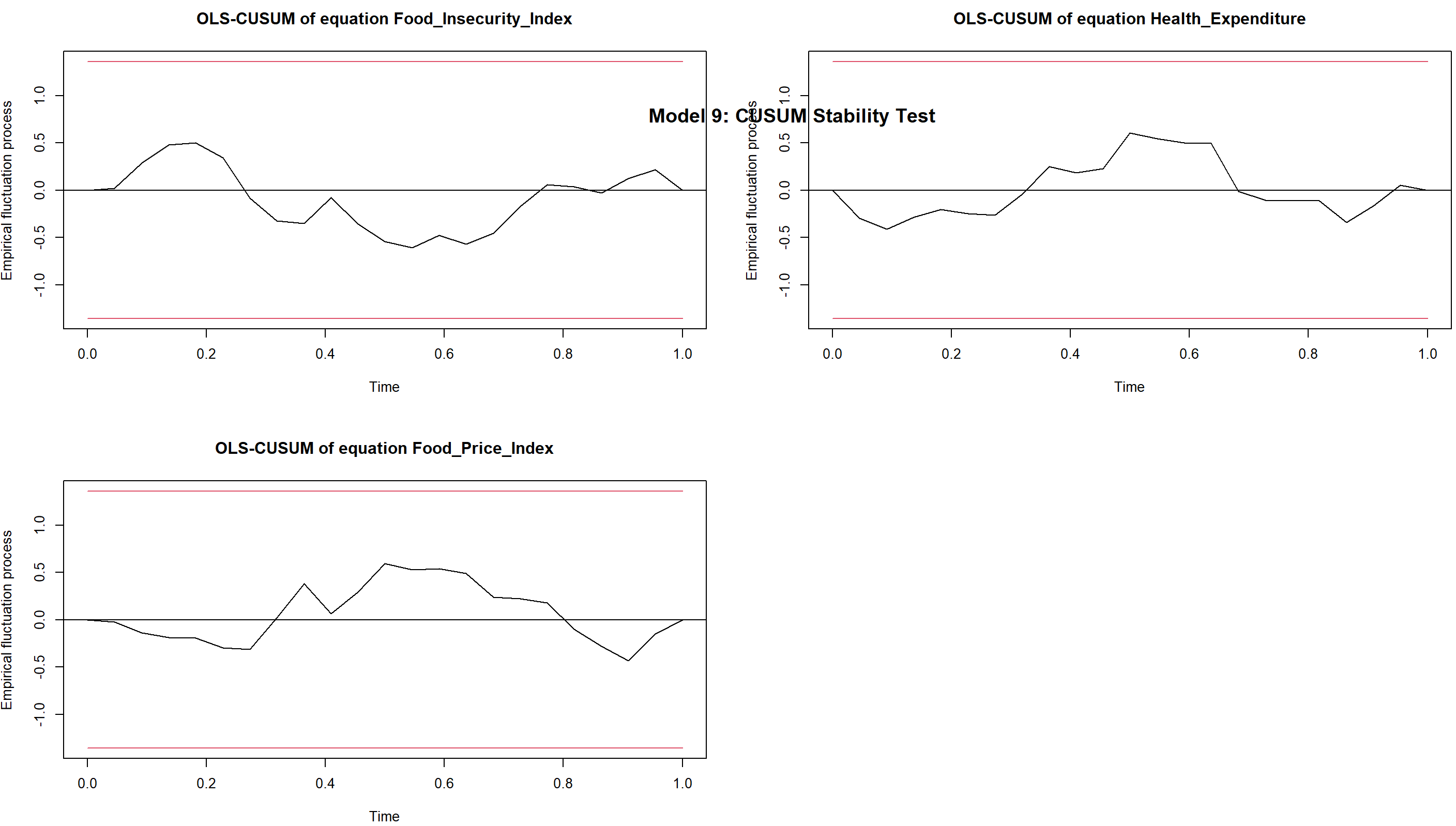 | | | 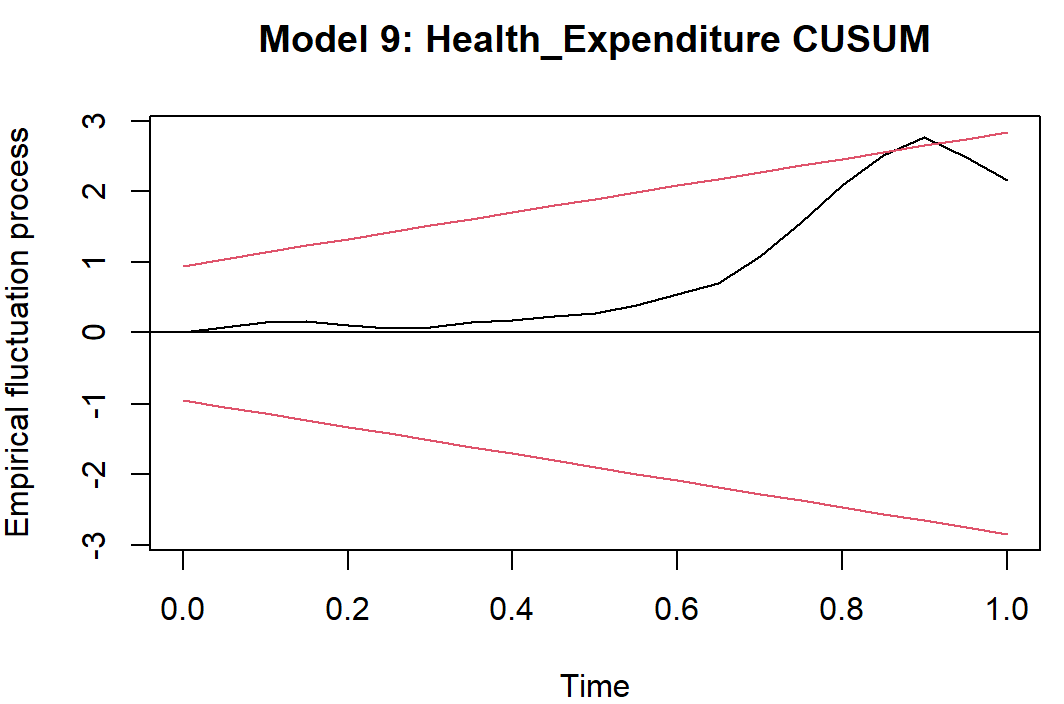 |
| 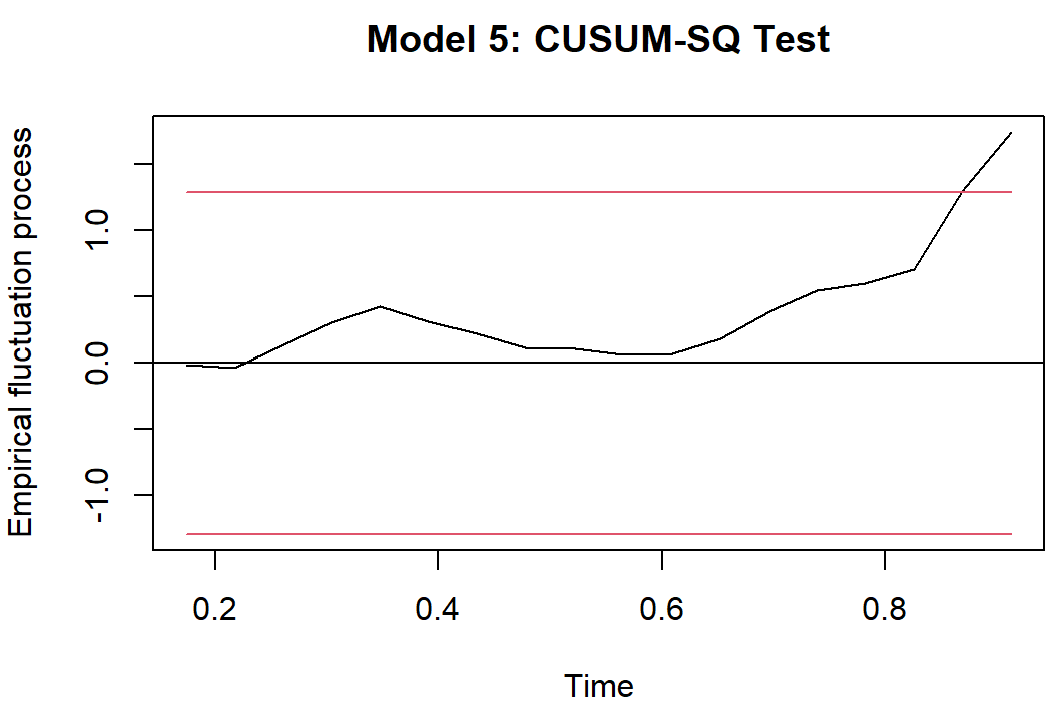 | 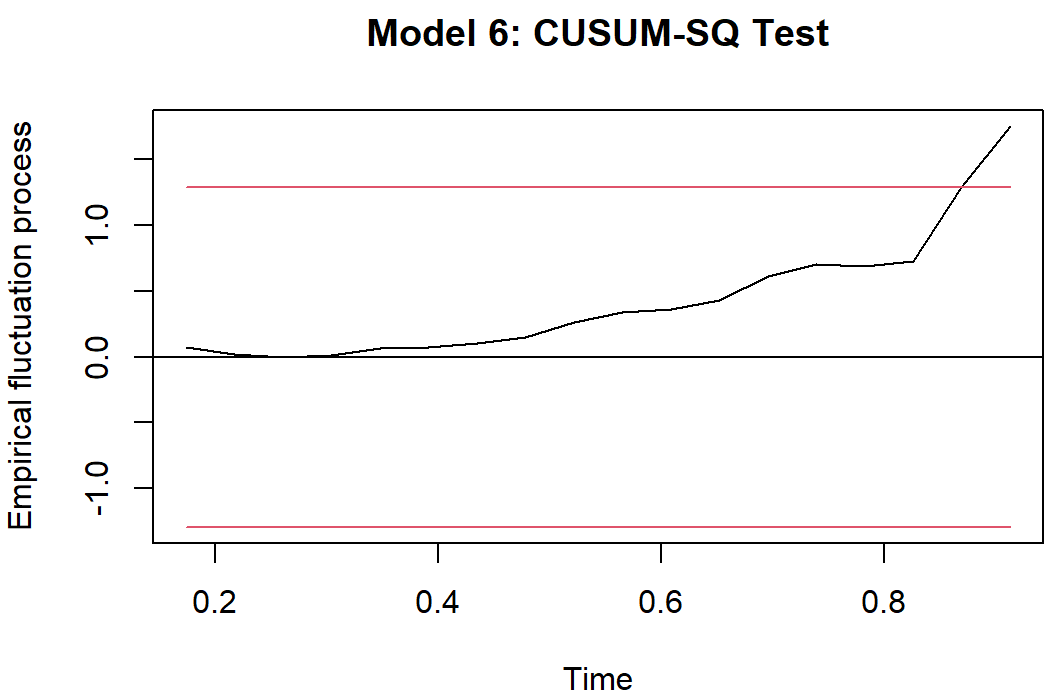 | | |
| 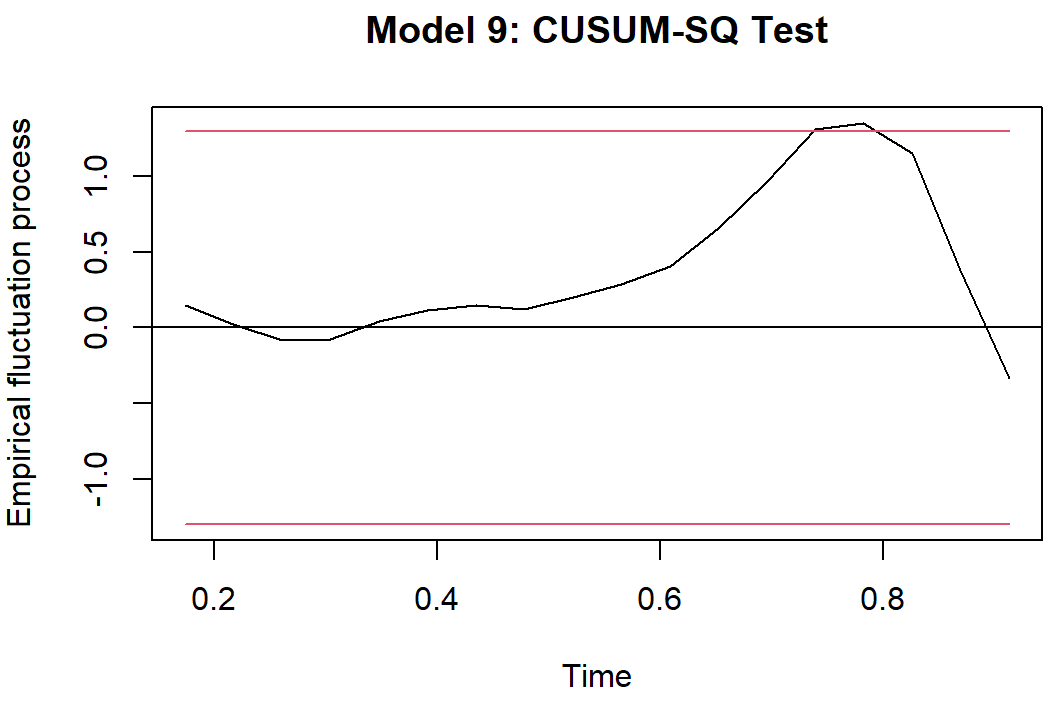 | | | |

Figure 1. Bai Perron Tests’, CUSUM and CUSUM SQ Tests’ Graphics

**Appendix B. Forecasting Health Expenditure for 2022 (Initial Submission Approach)**

In the initial submission of this manuscript, official data on Health Expenditure (HE) for the year 2022 had not yet been released by the World Bank. Therefore, in order to ensure continuity in the time series and preserve the minimum number of observations required for robust analysis, a one-year forecast was conducted using Holt’s linear trend method. This supplementary section documents the forecasting procedure and model diagnostics. Following the reviewer’s recommendation and the subsequent release of the actual 2022 data, the forecasted value was removed from the main analysis and replaced with the official figure. However, for transparency and methodological completeness, the initial forecasting approach is presented below.

A 1-year forecast ensured a sufficient number of observations for *HE*. Holt’s linear trend model was used to estimate *HE* for 2022. MAPE (2.99%) and RMSE (38.24) values were calculated to evaluate the model’s accuracy. Since the MAPE is below 10% and the RMSE is within an acceptable range, we concluded that the model demonstrates a strong predictive performance.

$$\iota_{t}={\alpha*y}_{t}+\left( 1-\alpha\right)*(\iota_{t-1}+b_{t-1})$$

$$b_{t}=\beta*\left( \iota_{t}-\iota_{t-1} \right)+\left( 1-\beta\right)*b_{t-1}$$

$$\hat{y_{t+m}}=\iota_{t}+m*b_{t}$$

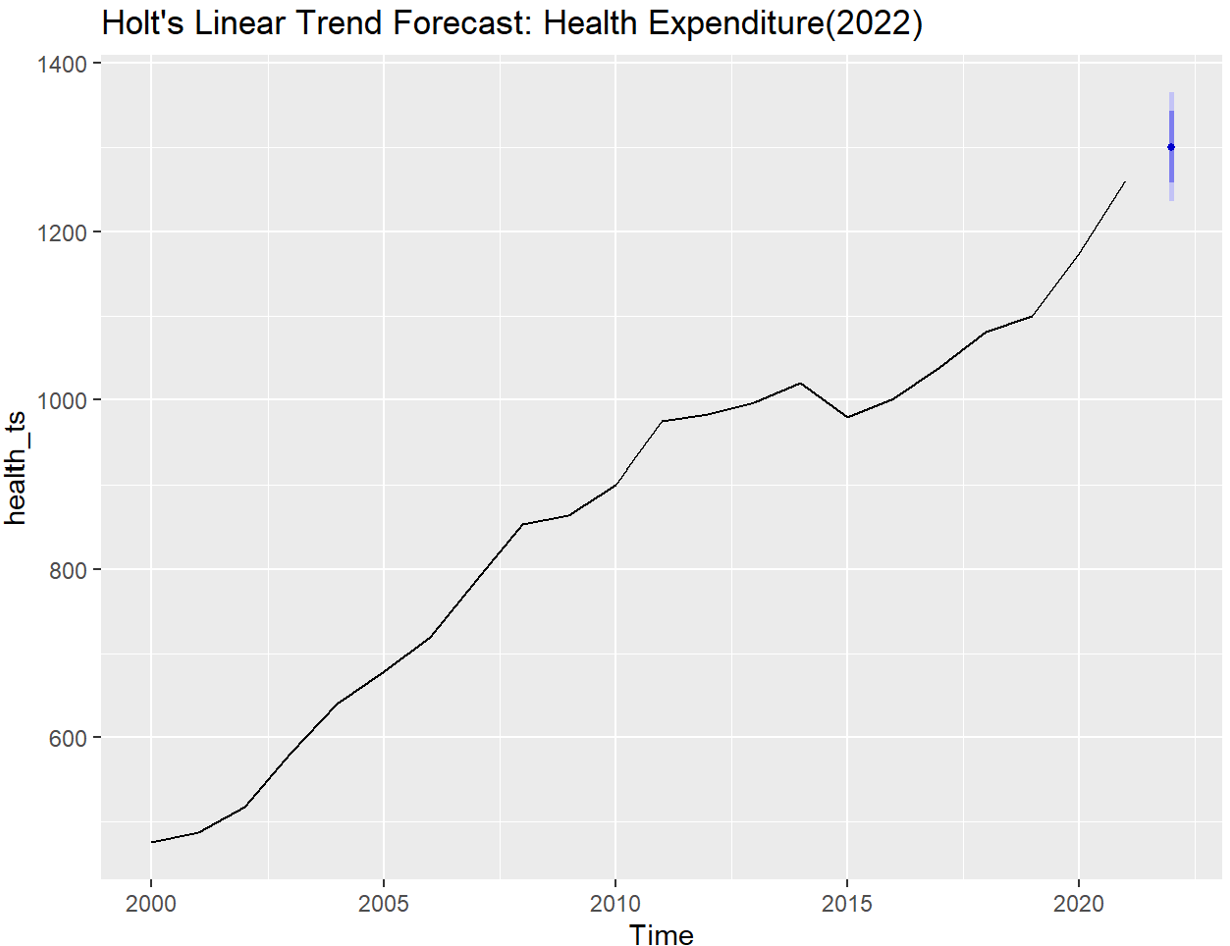


Figure 2. Estimation of Health Expenditure for the Year 2022

The model’s alignment with historical data is evident in the results presented in Figure 3, and the predicted value of **1300.78783** has been incorporated into the relevant section of the dataset. The obtained estimate indicates that **HE continued to increase consistently in line with the overall trend**.
